# AhR signaling in skin-resident CD207^+^ cells is involved in UV-B-induced amelioration of neuroinflammation

**DOI:** 10.1073/pnas.2424009122

**Published:** 2025-09-02

**Authors:** Nadine Mykicki, Johannes A. Eble, Heike Weighardt, Elena Gonnelli, Maria Schneeweiß, Charlotte Esser, Carsten Weishaupt, Maryam Rezaei, Irmgard Förster, Nicholas Schwab, Heinz Wiendl, Björn E. Clausen, Sven G. Meuth, Karin Loser

**Affiliations:** ^a^Department of Immunology, University of Oldenburg, Oldenburg 26129, Germany; ^b^Institute for Physiological Chemistry and Biochemistry, University of Münster, Münster 48149, Germany; ^c^LIMES–Life and Medical Science Institute, Bonn 53115, Germany; ^d^Department of Dermatology, Experimental Dermatology and Immunobiology of the Skin, University of Münster, Münster 48149, Germany; ^e^Leibniz Insitut Für Umweltmedizinische Forschung–Leibniz Research Institute for Environmental Medicine, Düsseldorf 40225, Germany; ^f^Department of Dermatology, University of Münster, Münster 48149, Germany; ^g^Department of Neurology, Institute for Translational Neurology, University of Münster, Münster 48149, Germany; ^h^Institute for Neurology and Neurophysiology, Faculty of Medicine, University of Freiburg, Freiburg 79106, Germany; ^i^Institute for Molecular Medicine and Research Center for Immunotherapy (Forschungszentrum Informatik), University Medical Center of the Johannes Gutenberg-University Mainz, Mainz 55131, Germany; ^j^Department of Neurology, Heinrich-Heine University Düsseldorf, Düsseldorf 40225, Germany

**Keywords:** multiple sclerosis, experimental autoimmune encephalomyelitis, UV-B, aryl hydrocarbon receptor, cutaneous dendritic cells

## Abstract

Environmental stimuli, including the exposure to ultraviolet (UV)-B light, are known to play a role in the modulation of immune-mediated mechanisms in multiple sclerosis (MS). In experimental autoimmune encephalomyelitis (EAE), we have shown that UV-B irradiation ameliorates disease outcome by regulatory T cells (Treg) expansion. Moreover, the UV-B-mediated induction of Treg numbers was also observed in MS. The aryl hydrocarbon receptor (AhR) can be activated by environmental factors including UV-B-induced photoproducts of tryptophan. Thus, we investigated the role of AhR during the transmission of UV-B irradiation. Therefore, wild-type (WT) and AhR-deficient mice (AhR^–/–^) were irradiated with UV-B light and immunized with myelin oligodendrocyte glycoprotein (MOG)-peptide. In WT mice it was shown that UV-B irradiation reduces EAE symptoms by Treg expansion. This effect was abrogated in animals with AhR deficiency. To better understand the underlying mechanisms of AhR regulation, we used mice with a deletion of AhR specifically in different subsets of antigen-presenting cells (APC) that have been shown to mediate the expansion of Treg. Interestingly, we could show that the AhR activation in murine cutaneous APC was sufficient to switch APC from a stimulatory into a regulatory phenotype, and moreover, responsible for APC cell maturation and migration into regional lymph nodes. Thus, our data indicate that AhR activation in APC might be required for UV-B-mediated immunosuppression during MOG-induced EAE. Hence, activation of AHR in tissue-resident APC, potentially by low-dose UV-B irradiation, might be beneficial as an adjuvant treatment in inflammatory or degenerative diseases of the central nervous system.

## Significance

Low sunlight exposure is associated with the susceptibility and severity of multiple sclerosis. Moreover, it has been shown that ultraviolet (UV)-B light causes immune cell alterations in human disease as well as increases levels of Treg. To analyze the beneficial effects of UV-B-light in the context of neuroinflammation, the mouse model of experimental autoimmune encephalomyelitis was used. We could show that aryl hydrocarbon receptor (AhR) activation by UV-B light in cutaneous antigen-presenting cells (APC) is not only responsible for switching APC from a stimulatory into a regulatory phenotype but also for APC cell maturation and migration into regional lymph nodes. Hence, this study suggests a central role for AhR signaling in UV-B-triggered immune regulation and protection from neuroinflammation.

Multiple sclerosis (MS) is an immune-mediated, chronic inflammatory and demyelinating disease of the central nervous system (CNS) leading to severe disability. Besides genetic susceptibility factors, environmental conditions including the exposure to ultraviolet (UV-B) light are known to play a role in the modulation of immune cell-mediated mechanisms in MS (1, 2). In experimental autoimmune encephalomyelitis (EAE), we demonstrated that irradiation with UV-B light ameliorated disease perpetuation (1). In this context, UV-B irradiation mediated the induction of regulatory T cells (Treg) that efficiently suppressed pathogenic T helper 1 (T_H_1) and T_H_17 responses. Moreover, in a small proof-of-concept study in MS patients, we have shown that UV-B light caused the alteration of immune cells in human disease since increased levels of Treg were detectable in peripheral blood after versus before local UV-B treatment of patients (1).

One mechanism of immune regulation in response to UV-B light is the activation of the transcription factor aryl hydrocarbon receptor (AhR). AhR is constitutively expressed in almost all cells of the skin and the CNS (3). Interestingly, endogenous ligands of AhR, such as 6-Formylindolo[3,2-b]carbazol (FICZ), which is derived from tryptophan upon UV-B irradiation, are known to control peripheral as well as central immune responses (4, 5). Accordingly, AhR activation ameliorated disease perpetuation and reduced the infiltration of pathogenic T cells into the CNS by expanding immunosuppressive Treg in EAE induced with myelin oligodendrocyte glycoprotein (MOG)-peptide (6, 7).

Moreover, AhR signaling is mediating UV-B-induced immunosuppression and controls the regulatory function of skin-resident antigen-presenting cells (APC) (8, 9).

Although AhR is highly expressed in barrier tissues like the skin and can be activated by environmental factors resulting in the modulation of systemic immune responses (10, 11), it is not yet fully understood how the activation of AhR by external stimuli, including UV-B irradiation, governs the outcome of systemic autoimmune diseases like EAE or MS. Here, we show that UV-B mediated AhR activation in cutaneous skin-resident Langerin^+^ cells is crucial for their maturation and migration into regional lymph nodes and therefore, for the expansion of immunosuppressive Treg, which finally ameliorated the outcome of EAE. Hence, with this study we provide evidence that AhR activation in APC is directly involved in UV-B-induced immune suppression in the context of neuroinflammation. Thus, interfering with AhR signaling in tissue-resident APC might potentially be useful as environment mimetic in the treatment of inflammatory and degenerative CNS disorders.

## Results

### UV-B-Induced Amelioration of EAE Is Abrogated in the Absence of AhR.

**Fig. 1.**
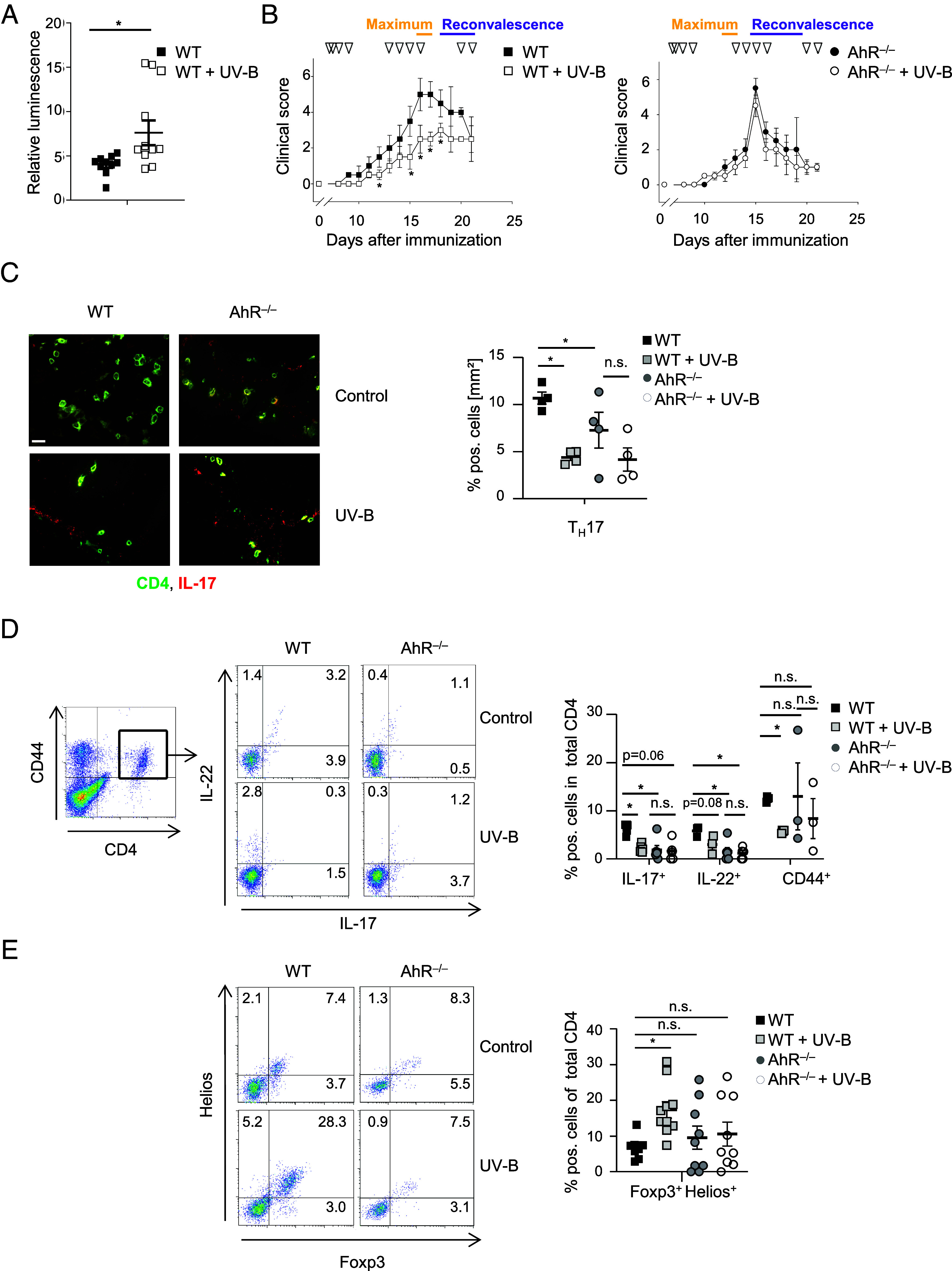
UV-B-induced amelioration of EAE is abrogated in the absence of AhR. Wild type (WT, n = 13 at disease maximum and n = 3 at reconvalescence phase) as well as AhR^–/–^ [n = 18 (disease maximum) and n = 8 (reconvalescence)] mice were immunized with MOG peptide and irradiated with UV-B light as described in the *Materials and Methods*. Triangles indicate the days of irradiation. (*A*) Serum samples were collected from irradiated WT mice and untreated controls at disease maximum. Hepa 1.6 cells were transfected with the indicated luciferase reporter plasmids and stimulated with serum samples. One day later, relative units of luminescence were determined as an indication for AhR ligand activity in the serum. (*B*) Mean clinical scores are shown. (*C*) Immunofluorescence staining of murine brain tissue from UV-B irradiated and nonirradiated WT and AhR^–/–^ mice at disease maximum. One representative image (*Left*) as well as the statistical evaluation from n = 4 mice out of three different experiments (*Right*) are shown. To evaluate T_H_17 numbers, cells were counted in nine visual fields of each cryosection. Original magnification 400×, (Scale bar, 20 µm.) (*D*) Flow cytometry of effector T cells in the CNS at disease maximum. Representative density plots (*Left*) and the statistical evaluation (*Right*) from n = 3 mice per group are shown. Cell subsets are gated for CD4^+^CD44^+^ T cells. (*E*) Flow cytometry of Treg in the CNS at disease maximum. Representative density plots (*Left*) and the statistical evaluation from n ≥ 9 mice per group (*Right*) are shown. *P < 0.05 (Mann–Whitney *U* test for *A* and *B*; One-way ANOVA for *C*–*E*. *P*-value was adjusted with Dunnett’s test for multiple comparisons). The data are shown as the means ± SEMs.

Previously, we have shown that UV-B light significantly delayed the onset of EAE symptoms and reduced disease severity. This effect was mediated by the induction of tolerogenic dendritic cells (DC) and Treg in the peripheral immune system that migrated to the CNS and down-regulated the activity of encephalitogenic T cells (1). Considering that it was previously shown that AhR signaling causes immunosuppression by modulating the phenotype of APC, we analyzed whether AhR activation might play a direct role for UV-B-induced immunosuppression during EAE (8, 12). UV-B-irradiated WT mice revealed significantly enhanced AhR ligand activity, which might be due to an increase of AhR ligands in the serum (Fig. 1*A*). Hence, we assumed that these compounds regulate AhR activation and therefore, the UV-B-mediated immune regulation.

To test this hypothesis, C57BL/6 (WT) and AhR^–/–^ mice were immunized with MOG peptide and irradiated with 150 mJ/cm^2^ UV-B light daily on four consecutive days, left untreated for 3 d, followed by a second irradiation period of 4 d and a 3-d treatment-free interval, because this regimen was shown to induce the most prominent immunosuppressive effects (1). Interestingly, whereas disease severity was reduced in WT mice upon UV-B irradiation as expected, the course of EAE was not altered in UV-B-irradiated AhR^–/–^ mice compared to nonirradiated controls (Fig. 1*B*). However, as shown by Veldhoen et al. AhR^–/–^ mice recovered faster after disease maximum and entered the phase of reconvalescence earlier than WT mice (Fig. 1*B*) (13).

Histological analyses at disease maximum (d15–d17 after immunization; *SI Appendix*, Fig. S1) showed fewer inflammatory foci and demyelinated areas in spinal cord sections from UV-B-treated compared to nonirradiated WT mice, thus confirming the amelioration of clinical symptoms. This UVB-mediated alleviation of EAE was absent in AhR^–/–^ mice showing comparable numbers of inflammatory foci and demyelinated areas in the CNS (*SI Appendix*, Fig. S1), indicating that AhR activation might play a role in UV-B-mediated immune regulation during EAE. It was shown that AhR is essential for T_H_17 cell differentiation (13). Therefore, we analyzed the numbers of T_H_17 cells in the CNS by immunofluorescence staining and flow cytometry analysis. Consistent to Veldhoen et al. and Quintana et al. (14, 15), frequencies of T_H_17 cells were reduced in brain tissues from AhR^–/–^ mice compared to WT mice (Fig. 1 *C* and *D*). Interestingly, flow cytometry analyses revealed significantly reduced CD44 levels in the CNS from UV-B-treated WT mice compared to nonirradiated controls (Fig. 1*D*). This effect was also absent in the CNS from AhR^–/–^ mice (Fig. 1*D*) suggesting an AhR-dependent reduced activation of T cells upon UV-B irradiation. Moreover, as shown in Fig. 1 *C* and *D*, the UV-B mediated reduction of T_H_17 cells was abrogated in AhR^–/–^ mice.

In MOG-induced EAE UV-B light exerts its immunomodulatory effects by the generation of tolerogenic DC in the skin, capable of expanding immunosuppressive Treg, which migrate to the CNS (1, 16, 17). Therefore, we quantified Treg numbers in the CNS at disease maximum (d15–d17 after immunization). Flow cytometry analyses (Fig. 1*E*) revealed that Foxp3^+^Helios^+^ cell numbers were not elevated in irradiated versus nonirradiated AhR^–/–^ mice, indicating that AhR activation by UV-B light might play a role during the expansion of Treg. This is in line with observations made by Navid et al., who revealed that AhR signaling is involved in UV radiation (UVR)-induced Treg expansion in murine contact hypersensitivity (CHS) (18).

The transcription factor Helios has been proposed to be differentially expressed between thymus-derived and peripherally induced Treg and, moreover, was associated with an improved suppressor function of these cells (19–21). As shown in Fig. 1*E*, upregulated levels of Foxp3^+^Helios^+^ cells were present in the CNS from irradiated WT mice, but not in AhR^–/–^ mice (Fig. 1*E*). This result suggests that UV-B light appears to expand and/or differentiate preexisting, thymus-derived Treg rather than induce Treg from conventional CD4^+^ T cells in the peripheral immune system.

To confirm the UV-B-induced activation of AhR signaling, AhR expressing cells were quantified by flow cytometry in skin-draining lymph nodes. Furthermore, the messenger RNA (mRNA) expression of the AhR related target genes cytochrome P450 family member A1 (*Cyp1a1*) and *Cyp1b1* was quantified in UV-B-irradiated and nonirradiated skin. Intriguingly, upon UV-B irradiation, the expression of AhR was upregulated in T_H_17 cells, Treg, and cutaneous Langerin^+^ cells in skin-draining lymph nodes (Gating strategy according to *SI Appendix*, Figs. S2 and S3*A*), further indicating that UV-B irradiation increases AhR signaling in the skin of MOG-immunized WT mice. In agreement, we detected a significantly enhanced expression of the AhR target genes *Cyp1a1* and *Cyp1b1* in UV-B-irradiated skin compared to nonirradiated WT controls (*SI Appendix*, Fig. S3*B*). As expected, AhR expressing T cells and APC were absent in AhR^–/–^ mice (*SI Appendix*, Fig. S3*A*) and consequently, UV-B light was not able to modulate the expression of AhR target genes (*SI Appendix*, Fig. S3*B*). One negative regulatory mechanism of AhR-driven gene expression is the AhR repressor (AhRR), which competes with AhR for binding to ARNT (22, 23). Since *Ahrr* gene expression was comparable in all groups (*SI Appendix*, Fig. S3*B*) it might be conceivable that UV-B irradiation upregulates AhR without simultaneously increasing the expression of its inhibitor *Ahrr* or that the kinetics of Cyps and Ahrr mRNA expression might be differently regulated. Thus, further pointing toward a role of AhR signaling in response to UV-B light.

### AhR Activation in CD11c^+^ or CD207^+^ Cutaneous APC Is Crucial for UV-B-Induced Amelioration of EAE.

We have previously shown that UV-B expands Foxp3^+^ Treg via the induction of tolerogenic DC in the skin (16, 17). This is in line with observations made by Quintana et al. and Kenison-White et al., who reported that the AhR ligand [2-(1′H-indole-3′-carbonyl)-thiazole-4-carboxylic acid methyl ester (ITE)] induces tolerogenic DCs in the spleen, which promotes FoxP3^+^ Treg differentiation as well as type 1 regulatory T cells (Tr1 cells), and, finally, EAE suppression (12, 24). Hence, we analyzed whether AhR activation in APC might be essential for the UV-B-induced amelioration of EAE and therefore, deleted AhR in different APC subsets.

**Fig. 2.**
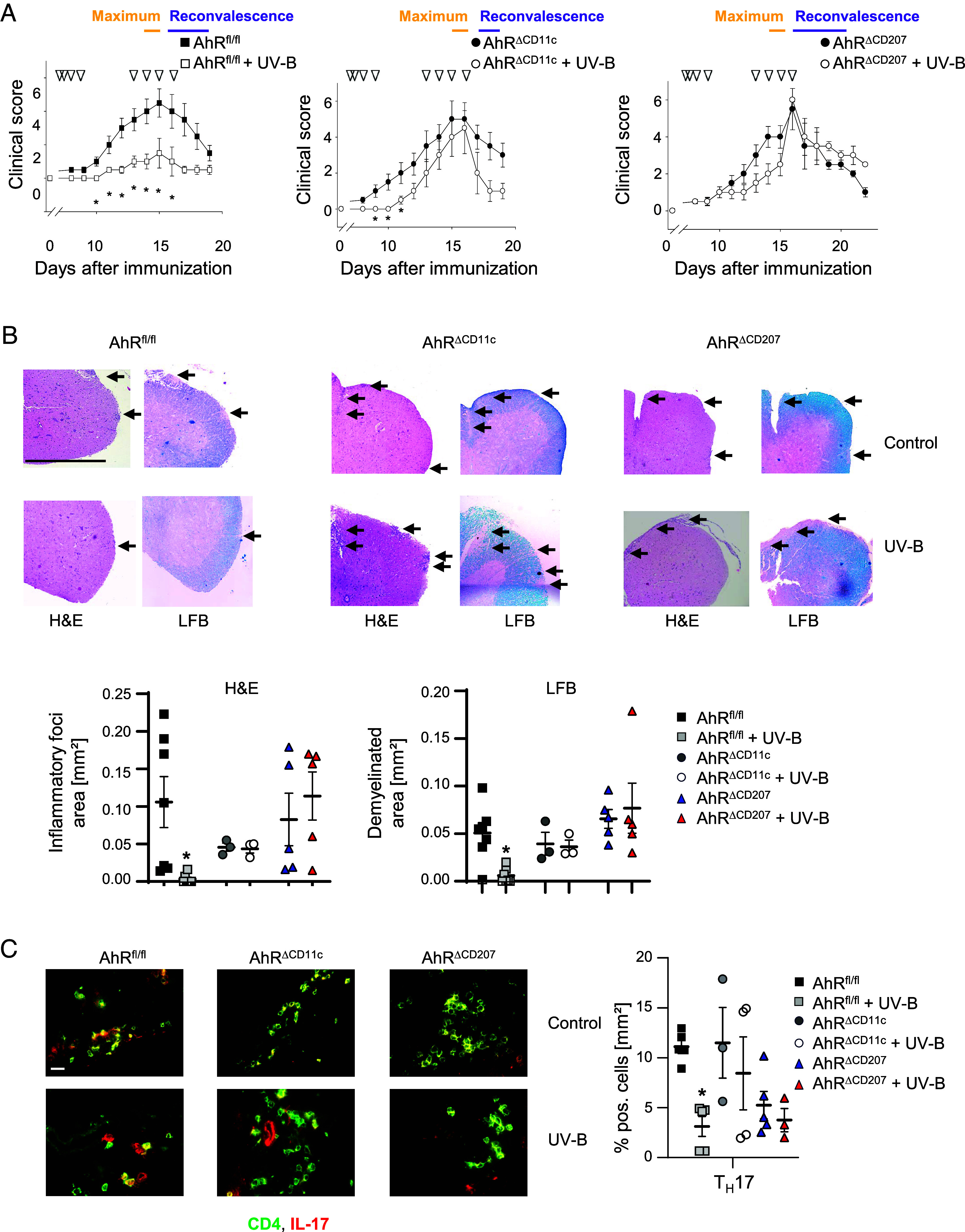
AhR activation in CD11c^+^ or CD207^+^ cutaneous APC is crucial for UV-B-induced amelioration of EAE. AhR^fl/fl^ (*n* = 11 at disease maximum)/*n* = 4 at reconvalescence), AhR^ΔCD11c^ (*n* = 10 at disease maximum/ *n* = 3 at reconvalescence) as well as AhR^ΔCD207^ (*n* = 11 at disease maximum, *n* = 3 at reconvalescence) mice were immunized with MOG peptide and irradiated with UV-B light as described in the *Materials and Methods*. Triangles indicate the days of irradiation. (*A*) Mean clinical scores are shown. (*B*) Representative H&E as well as LFB staining in lumbal spinal cord at disease maximum as well as statistical analyses of inflammatory foci and demyelinated areas from *n* ≥ 3 mice from three different experiments are shown. Original magnification 100×, (Scale bar, 200 µm.) Infiltration of mononuclear cells and demyelinated areas are indicated by arrows. (*C*) Immunofluorescence staining of murine brain tissue from UV-B irradiated and nonirradiated AhR^fl/fl^, AhR^ΔCD11c^, and AhR^ΔCD207^ mice at disease maximum using antibodies against CD4 (green) and IL-17A (red). One representative image (*Left*) as well as the statistical evaluation from *n* ≥ 3 mice out of three different experiments (*Right*) are shown. To evaluate T_H_17 numbers, cells were counted in nine visual fields of each cryosection. Original magnification 400×, (Scale bar, 20 µm.) *P < 0.05 (Mann–Whitney *U* test for *A*; One-way ANOVA for *B* and *C*. *P*-value was adjusted with Tukey’s (*B*) or Sidak’s (*C*) test for multiple comparisons). The data are shown as the means ± SEMs.

Interestingly, UV-B-irradiation failed to ameliorate EAE severity in mice specifically lacking AhR in CD11c^+^ (AhR^ΔCD11c^) or selectively in CD207^+^ cutaneous APC (AhR^ΔCD207^) (Fig. 2*A*). Of note, UV-B treatment led to a delayed onset of EAE symptoms and a reduced disease severity in AhR^fl/fl^ control mice (Fig. 2*A*). In accordance with the clinical symptoms, histological analysis at disease maximum (d15–d17 after immunization, Fig. 2*B*) showed similar numbers of inflammatory foci and demyelinated areas in lumbal spinal cord tissue from UV-B-irradiated AhR^ΔCD11c^ as well as AhR^ΔCD207^ mice compared to nonirradiated controls, indicating that AhR signaling, in particular (cutaneous) APC subsets might indeed be critical for the beneficial effect of UV-B in MOG-immunized mice. Since CD207^+^ APC are able to express CD11c upon activation and maturation (25, 26) and can be found in the epidermis and upper dermis which characterizes them as primary cell subset encountering the environmental stimulus UV-B light, it is highly likely that specifically AhR activation in CD207^+^ cutaneous APC was crucial for UV-B-mediated amelioration of EAE symptoms.

### UV-B Light Fails to Induce Immunosuppressive Immune Cells in AhR^ΔCD11c^ and AhR^ΔCD207^ Mice.

**Fig. 3.**
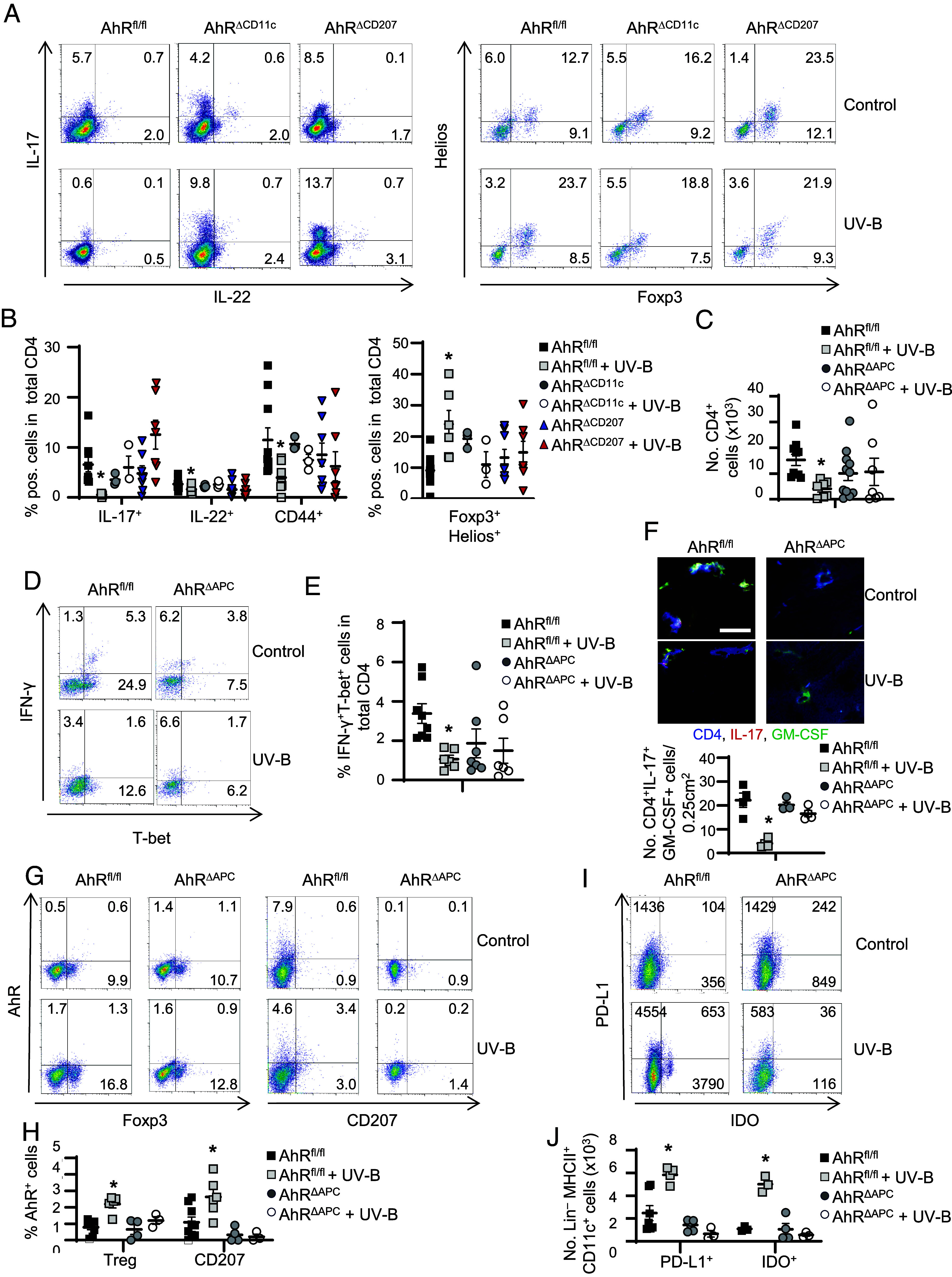
UV-B light fails to induce immunosuppressive immune cells in AhR^ΔCD11c^ and AhR^ΔCD207^ mice. (*A* and *B*) Flow cytometry of pathogenic T cells (*Left*) and Treg (*Right*) in the CNS at disease maximum. Representative density plots (*A*) and statistics (*B*) from *n* ≥ 3 mice per group are shown; IL-17^+^ and IL-22^+^ cells were gated for CD4^+^CD44^+^ T cells. (*C*) Flow cytometry of total CD4^+^ T cells in the CNS at disease maximum. Statistics from n ≥ 7 mice per group are shown. (*D* and *E*) Flow cytometry of T_H_1 cells in the CNS at disease maximum. Representative density plots (*D*) and statistics (*E*) from *n* ≥ 6 mice per group are shown; IFN-γ^+^ and T-bet^+^ cells were gated for CD4^+^CD44^+^ T cells. (*F*) Immunofluorescence staining of murine brain tissue from UV-B irradiated and nonirradiated AhR^fl/fl^ and AhR^ΔAPC^ mice at disease maximum using antibodies against CD4 (blue), IL-17A (red), and GM-CSF (green). One representative image and statistics (below) from *n* = 4 mice are shown. To evaluate CD4^+^IL-17^+^GM-CSF^+^ cell numbers, cells were counted in 12 visual fields of each cryosection. Original magnification 400×, (Scale bar, 20 µm.) (*P < 0.05 versus nonirradiated controls tested with One-way ANOVA. *P*-value was adjusted with Tukey’s test for multiple comparisons). (*G* and *H*) Flow cytometry to quantify AhR expression in Treg or CD207^+^ APC in skin draining lymph nodes at the disease maximum. Representative density plots (*G*) and the statistical evaluation from *n* ≥ 4 mice per group (*H*) are shown. (*I* and *J*) Flow cytometry showing PD-L1 and IDO suppressing MHC^+^CD11c^+^ cells in skin draining lymph nodes at the disease maximum. Representative density plots (*I*) and the statistical evaluation from *n* ≥ 3 mice per group (*J*) are shown. *P < 0.05 versus nonirradiated controls tested with One-way ANOVA for *B*, *C*, *E*, *H*, and *J*. *P*-value was adjusted with Dunnett’s test for multiple comparisons.

To investigate, whether the UV-B mediated activation of AhR in all CD11c^+^ (AhR^ΔCD11c^ mice) or selectively in CD207^+^ cutaneous APC (as a subset of CD11c^+^; AhR^ΔCD207^ mice) was sufficient for Treg induction and thus, inhibition of encephalitogenic T cell activation, Treg, T_H_17, and T_H_1 cells infiltrating into the CNS were characterized by immunofluorescence staining and flow cytometry analyses. As shown in Fig. 2*C*, the UVB-mediated modulation in WT mice of T_H_17 cell was abrogated in mice with a specific deletion of AhR in CD11c^+^ (AhR^ΔCD11c^) or in CD207^+^ cutaneous APC (AhR^ΔCD207^). This effect was further confirmed by flow cytometric analyses at disease maximum (d15 – d17 after immunization) revealing neither altered numbers of CD44^+^ activated T cells as well as IL-17 producing CD4^+^CD44^+^ T cells nor Foxp3^+^Helios^+^ Treg in the CNS from mice with a specific deletion in myeloid cell subsets (AhR^ΔCD11c^ and AhR^ΔCD207^) upon UV-B irradiation (Fig. 3 *A*–*C*). Additionally, as shown in Fig. 3 *C*–*F* and *SI Appendix*, Fig. S4 *A* and *B*, total numbers of CD4^+^ T cells, T_H_1 cells as well as of CD4^+^ T cells coexpressing IL-17 and GM-CSF or IFN-γ infiltrating into the CNS were decreased in UV-irradiated compared to nonirradiated AhR^fl/fl^ mice but not in the CNS of mice with a cell-type specific deletion of AhR in APC.

Expectedly, whereas AhR expression was upregulated by UV-B light in Treg and APC in lymph node cells from AhR^fl/fl^ compared to nonirradiated controls, this effect was not observable in mice lacking AhR in APC (Fig. 3 *G* and *H*; gating strategy see *SI Appendix*, Fig. S2), again supporting the hypothesis that AhR activation especially in tissue-resident Langerin^+^ APC, might be essential for the protective and immunosuppressive effect of the environmental stimulus UV-B light in MOG-induced EAE.

To investigate the impact of UV-B induced AhR activation on IL-10 production in Treg and non-Treg, we quantified IL-10 in different cell subsets isolated from the CNS of irradiated and control mice. As shown in *SI Appendix*, Fig. S5 *A* and *B*, UV-B irradiation led to markedly increased concentrations of IL-10 in Foxp3^+^ but not in Foxp3^–^ T cells from AhR^fl/fl^ mice. This effect was not detectable in mice with a cell specific deletion of AhR in cutaneous APC (*SI Appendix*, Fig. S5*A*). Moreover, in non-T cells UV-B irradiation slightly upregulated IL-10 levels, which might be related to the induction of immunosuppressive tolerogenic DC (*SI Appendix*, Fig. S5 *C* and *D*). Thus, it is highly likely that Foxp3^+^ Treg as well as tolerogenic DC were responsible for the IL-10 mediated effects of UV-B.

To better understand whether the UV-B-induced upregulation of AhR expression in CD11c^+^ or particularly CD207^+^ APC might indeed alter their phenotype and function, we purified these cell subsets from skin draining lymph nodes of UV-B irradiated EAE mice and nonirradiated controls. Subsequently, we analyzed their phenotype by quantifying the expression of markers characteristic for tolerance, since it was shown previously that UV-B irradiation has the potential to induce tolerogenic DC, which are crucial for the induction of Treg (1, 8). In line with these publications, we detected reduced numbers of cells expressing the coinhibitory ligand PD-L1 or indoleamine-2,3-dioxygenase (IDO), the central enzyme in tryptophan catabolism in the lymph nodes of irradiated and nonirradiated mice specifically lacking AhR in APC (Fig. 3 *I* and *J*), which might explain the reduced Treg expansion in LN and CNS upon UV-B irradiation in mice with an AhR deletion in APC (Fig. 3 *A* and *B*).

### Langerin^+^ Cell Maturation and Migration Are Impaired in AhR-Deficient Mice.

**Fig. 4.**
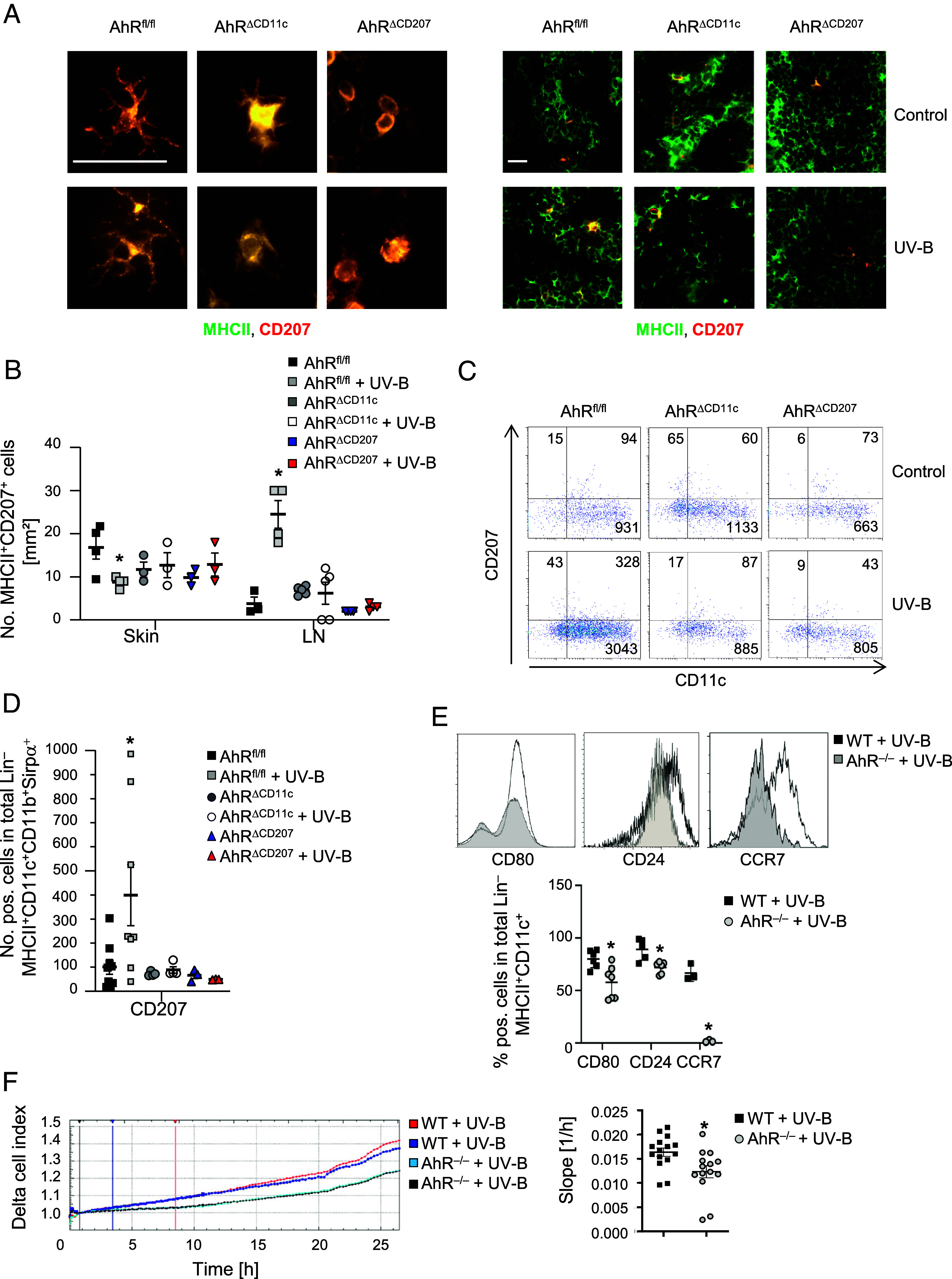
Langerin^+^ cell maturation and migration are impaired in AhR-deficient mice. (*A* and *B*) Immunofluorescence staining of murine epidermal sheets (*Left*) as well as from skin draining lymph nodes (*Right*) from UV-B irradiated and nonirradiated AhR^fl/fl^, AhR^ΔCD11c^, and AhR^ΔCD207^ mice at disease maximum. One representative image (*A*) as well as the statistical evaluation (*B*) from *n* ≥ 3 mice as of three different experiments are shown. To evaluate cell numbers, cells were counted in five visual fields of each cryosection. Original magnification 400×, (Scale bar, 20 µm.) (*C*) Flow cytometry of CD207^+^ cells in skin draining lymph nodes at disease maximum. Representative density plots and statistics (*D*) from *n* = 3 mice per group are shown; cells are gated for Lin^–^MHCII^+^CD11c^+^CD11b^+^Sirpα^+^. (*E*) Flow cytometry of costimulatory molecules in BM-DC of WT (black curve) and AhR^−/−^ (gray curve) mice after UV-B stimulation. Representative Overlay (*Top*) and statistics (*Bottom*) from *n* ≥ 3 mice per group are shown; cells are gated for Lin^–^MHCII^+^CD11c^+^. (*F*) Migration assay of UV-B-irradiated BM-DC of WT versus AhR^−/−^ mice. Cells were cultured in the presence of GM-CSF and IL-4 and stimulated with 1.25 mJ/cm^2^ UV-B. Cells were seeded on a CIM plate coated with 10 μg/mL bovine Collagen-I. Cell migration was measured as change of impedance and read out as Delta cell index. Migration rates are measured as slopes of delta cell index values over a time period (marked by blue and red vertical lines in (*F*). Representative migration of BM-DC of 2 WT and AhR^−/−^ mice (*Left*) as well as statistics (*Right*) during 5 h of measurement from *n* = 3 mice and 14 to 15 replicates are represented. *P < 0.05 (Student’s *t* test for *E*; Mann–Whitney *U* test for *F*; One-way ANOVA for *B*, *D*, and *E*. *P*-value was adjusted with Dunnett’s test for multiple comparisons). The data are shown as the means ± SEMs.

**Fig. 5.**
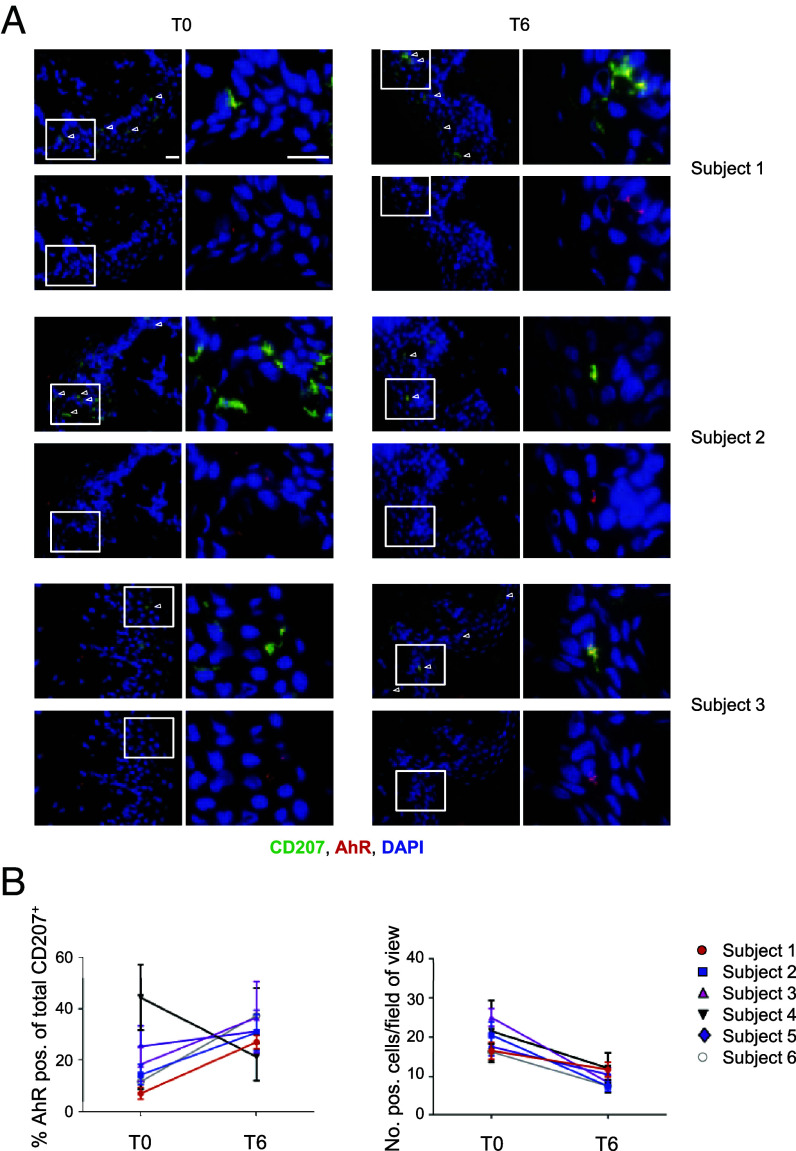
AhR upregulation in CD207+ cells in MS patients after 6 wk of UV-B irradiation. (*A* and *B*) Immunofluorescence staining of human skin from MS patients before (T0) and 6 wk after phototherapy (T6). Three representative images (*A*) as well as the statistical evaluation (*B*) from *n* = 6 individuals are shown. To evaluate the numbers of total CD207^+^ and AhR^+^CD207^+^ cells, cells were counted in six visible fields of each cryosection. Original magnification 200×, (Scale bar, 20 µm.)

Breuer et al. identified that epidermal Langerhans cells (LC) are critically involved in the generation of UV-B induced Treg (1). To elucidate the mechanism associated with UV-B-mediated activation of AhR in cutaneous APC during MOG-induced EAE, we analyzed Langerin^+^ cells in detail. Fluorescence microscopy of epidermal sheets and skin tissue from mice with a specific deletion in CD11c^+^ mature (AhR^ΔCD11c^) as well as in CD207^+^ cutaneous DC AhR^ΔCD207^ did not reveal any differences in numbers of LC per mm^2^ (Fig. 4 *A* and *B*). However, the ramification of LC dendrites especially in epidermal skin sheets from AhR^ΔCD207^ mice revealed striking changes (Fig. 4*A* and *SI Appendix*, Fig. S4*C*) indicating a lower granularity and/or an impaired maturation in these mice. However, numbers of LC per mm^2^ were reduced in epidermal sheets from UV-B-irradiated AhR^fl/fl^ mice compared to nonirradiated controls suggesting an improved migration behavior of Langerin^+^ cells to the skin draining lymph nodes upon UV-B treatment (Fig. 4*B*). Therefore, we analyzed the numbers of Langerin^+^ cells in the skin draining lymph nodes by immunofluorescence staining (in the paracortical structure; see *SI Appendix*, Fig. S4*D*) and flow cytometric analyses. Indeed, we detected significantly increased numbers of CD207^+^ cells in skin draining lymph nodes from UV-B irradiated WT mice compared to nonirradiated controls (Fig. 4 *A* and *D*). In contrast, no significant changes concerning the numbers in the skin draining lymph nodes were observable from irradiated and nonirradiated AhR^ΔCD11c^ as well as AhR^ΔCD207^ mice (Fig. 4 *A* and *D*; gating strategy for LC see *SI Appendix*, Fig. S2). To verify, whether reduced numbers of AhR-deficient skin-migrating APC in the skin-draining lymph nodes were related to a reduced migration rate, we stimulated bone marrow–derived DC (BM-DC) either with LPS or with 1.25 mJ/cm^2^ UV-B irradiation and analyzed the expression of costimulatory molecules as well as the migration behavior. Strikingly, AhR-deficient APC not only remained smaller and less granular during UV-B irradiation (Fig. 4*A*), but also did not upregulate the expression of costimulatory molecules like CD80 and CD24 as well as the C-C- chemokine receptor type 7 (CCR7) during in vitro maturation (Fig. 4*E*) as it was shown for CHS (18, 27). Since the DC maturation process enhances their migratory capacity by upregulation of the lymph node homing receptor CCR7 (28) and CCR7 expression was reduced in BM-DC from AhR^–/–^ mice after UV-B treatment (Fig. 4*E*), AhR-deficient BM-DC showed a reduced migration after LPS (*SI Appendix*, Fig. S4*E*) or UV-B (Fig. 5*F*) stimulation in an in vitro assay. Hence, these data suggest that the protective effect of UV-B during EAE perpetuation was most likely mediated by AhR activation in cutaneous APC, followed by an increased migration of APC with a tolerogenic phenotype to the regional lymph nodes. In the lymph nodes tolerogenic APC are able to promote Treg expansion. Finally, these Treg have the ability to suppress pathogenic effector cells and disease symptoms. Cutaneous APC lacking AhR did not develop a tolerogenic phenotype after UV-B irradiation and showed an impaired migratory capacity, finally resulting in a reduced ability to induce Treg in regional lymph nodes (*SI Appendix*, Fig. S9).

However, it is known that the administration of complete Freund’s adjuvant (CFA) contained in the MOG-solution might activate Toll-like receptor signaling, thus resulting in immune cell activation. To exclude potential adjuvant effects, we investigated the effects of UV-B irradiation in a second, spontaneous model of EAE. Hence, TCR_MOG_ x IgH_MOG_ mice (Devic) (29), which spontaneously develop hind limb paralysis at the age of 5 to 6 wk were treated with UV-B light starting at the age of 21 d after birth. UV-B irradiated Devic mice showed a trend toward reduced disease severity compared to nonirradiated controls (*SI Appendix*, Fig. S6*A*). Moreover, upon UV-B irradiation we observed an enhanced mRNA expression of the Ahr target genes *Cyp1a1*, *Cy1b1,* and *Ido1* in the skin of Devic mice (*SI Appendix*, Fig. S6*B*), clearly indicating that UV-B irradiation upregulated AhR signaling. In addition to clinical signs, UV-B irradiation reduced the infiltration of pathogenic effector CD4^+^ T-cells expressing IL-17 together with IFN-γ or GM-CSF into the spinal cord (*SI Appendix*, Fig. S6*C*). Together, our observations in mice spontaneously developing EAE clearly show that the beneficial effects of low-dose UV-B irradiation were mediated by AhR activation and seemed to be independent of CFA administration.

### AhR Deletion in T Cells Has a Minor Effect on UV-B Mediated Amelioration of EAE.

Quintana et al. demonstrated that AhR signaling has opposite roles on T_H_17 and Treg cell differentiation in a ligand-specific manner (14). Since UV-B treatment upregulated AhR expression in T_H_17 cells as well as in Treg (*SI Appendix*, Fig. S3*A*), we asked whether UV-B-mediated AhR regulation in T cells might be crucial for the beneficial effects of UV-B light. Therefore, we irradiated and immunized mice lacking AhR in T cells (AhR^ΔLck^ mice).

Interestingly, UV-B-irradiation had a minor and not significant effect on EAE perpetuation in mice specifically lacking AhR in T cells (AhR^ΔLck^) (*SI Appendix*, Fig. S7*A*). Additionally, 30 to 40% of these mice developed no EAE symptoms at all. Of note, UV-B treatment led to a significantly reduced disease severity in AhR^fl/fl^ control mice (*SI Appendix*, Fig. S7*A*). Besides APC, Treg as well as other T cell subsets such as memory CD4^+^ T cells are expanded in the skin upon UV-B exposure (30, 31). Hence, it might be conceivable that UV-B irradiation has a direct effect on T cell subsets in the skin via activation of AhR. To investigate, whether the UV-B mediated activation of AhR in T cells was sufficient for Treg induction and thus, inhibition of encephalitogenic T cell activation, Treg and T_H_17 cells infiltrating into the CNS were characterized by flow cytometry at disease maximum (d15–d17 after immunization). As shown in *SI Appendix*, Fig. S7 *B* and *C* IL-17, IL-22 as well as CD44 levels were not altered in the CNS from irradiated and nonirradiated mice with an AhR specific deletion in T cells (AhR^ΔLCK^) presumably due to a failed induction of Foxp3^+^Helios^+^ Treg in these mice (*SI Appendix*, Fig. S7 *B* and *C*). These data strengthen the hypothesis that AhR activation in cutaneous T cells might have a minor but still observable effect for the UV-B-mediated immunosuppression. Additionally, these results were in line with Quintana et al., who identified that AhR is a regulator of Treg differentiation (14).

Worth mentioning, that the deletion of AhR in neurons (AhR^ΔCamkIIα^ mice) had no impact on the UV-B-induced protection from EAE (*SI Appendix*, Fig. S8).

### AhR Upregulation in CD207^+^ Cells in MS Patients after 6 wk of UV-B Irradiation.

Finally, we assessed whether UV-B irradiation might up-regulate AhR signaling in CD207^+^ cutaneous APC from MS patients as it was shown in the mouse model. Hence, the expression of CD207 and AhR was quantified in skin biopsies (taken before and after the UV-B phototherapy) from individuals with MS (1). Intriguingly, in line with the observations that we made in MOG-induced EAE, numbers of CD207^+^ cells were reduced after 6 wk of UV-B irradiation in every individual with MS indicating that UV-B triggered APC activation and migration into the lymph nodes in humans as well (Fig. 5 *A* and *B*). In line with this, the percentage of AhR expressing CD207^+^ cells was increased after UV-B irradiation in 5 out of 6 individuals with MS (Fig. 5 *A* and *B*). Hence, our data suggest that MS patients might benefit from AhR activation in cutaneous APC as well. Finally, these CD207^+^ cells could have entered regional lymph nodes and induced the generation of Treg, which would be in accordance with the increased Treg numbers that we detected in peripheral blood of these MS patients after phototherapy (1).

## Discussion

Here, we demonstrated that AhR activation in cutaneous CD207^+^ APC was required for UV-B-induced immunosuppression during MOG-induced EAE. Mechanistically, UV-B irradiation activated AhR in APC in the skin, followed by an increased maturation, lymph node homing, and migration of cutaneous tolerogenic APC and consequently, the ability to potentially induce immunosuppressive Treg, which finally resulted in the subsequent attenuation of disease symptoms (*SI Appendix*, Fig. S9).

Targeting AhR to control (auto)inflammation has frequently been discussed. One common mechanism of how AhR signaling regulates the immune response during EAE is that AhR ligands such as photoproducts of tryptophan, e.g. FICZ, induce receptor activation followed by tolerogenic APC induction, which finally promotes FoxP3^+^ Treg generation, shifting the immune balance from a pathogenic phenotype toward immune tolerance in the periphery and the CNS (4, 5, 7, 32). Additionally, AhR signaling modulates Treg differentiation by influencing the state of FoxP3 as well as IL-17 promoter methylation. On one site, this results in enhanced FoxP3 and on the other site, decreased IL-17 expression (33). Besides, AhR is not exclusively regulated within immune cells, but also in other cell types, such as human endothelial cells (34). Consequently, environmental stimuli like cigarette smoke or UV light, which are known to contain or generate AhR ligands, were shown to modulate the progression of MS (1, 35–37). Especially, the influence of UV light on the immune system and the connection between skin and CNS has been studied in the context of environmental stimuli relevant for CNS autoinflammation (1, 38, 39). In that relation, AhR was already recognized as a molecular target for UVR-exerting immunosuppression since blocking of AhR signaling with a specific antagonist reduced UVR-induced immunosuppression and AhR^–/–^ mice showed a reduced immunosuppressive response to UVR for instance during CHS (8, 18). Accordingly, UV-B stimulation failed to ameliorate EAE in the absence of AhR, mediated by an abolished induction of immunosuppressive cell subsets (Fig. 1). Here, we described AhR signaling in cutaneous APC as being responsible for UV-B mediated immunosuppression and amelioration of disease symptoms during EAE.

LC are important APC in the skin, where they recognize foreign and self-antigens resulting in maturation and migration into lymphoid organs, and finally, in T cell activation and differentiation. Hence, we hypothesized that in particular AhR activation in cutaneous APC e.g., CD207^+^ cells, would be essential for UV-B-mediated immunosuppression. Accordingly, Breuer et al. have discussed that LC are necessary mediators for systemic UV-B-induced immune regulation during EAE perpetuation since ablation of LC abolished this effect (1). Moreover, AhR plays a crucial role in LC maturation leading to a tolerogenic phenotype and therefore an impaired CHS in mice (8, 27). Jux et al. hypothesized that AhR deficiency in keratinocytes reduced GM-CSF secretion and therefore the expression of costimulatory molecules in LC, since GM-CSF addition to AhR^–/–^ LC rescued CD80 expression (27). However, GM-CSF supplementation induced CD80 expression in WT LC as well, resulting in a significant higher expression of the costimulatory molecule than in AhR^−/−^ LC (27). This result and the fact that the morphological changes of AhR^−/−^ persisted upon GM-CSF cultivation (27) indicated that other factors, which are relevant for LC maturation, are lacking in AhR^−/−^ mice as well. This was corroborated by the fact that already the specific deletion of AhR in CD207^+^ cells with unaffected keratinocytes resulted in failure of modulation of Treg or T_H_17 numbers and therefore to an abolished amelioration of EAE upon UV-B irradiation (Figs. 2 and 3). Bruhs et al. indicated that the release of IL-2 was functionally relevant for the induction of Treg by DC (8). In line with Jux et al. APC with an AhR deletion were smaller with lower granularity (Fig. 4*A*) and showed an impaired maturation (Figs. 3 *I* and *J* and 4 *D* and *E*) despite GM-CSF supplementation into BM-DC cultures indicating that a reduced number of motile LC migrate into the lymph nodes and stimulate T cell responses (Fig. 4 *B* and *C*). Although migration of irradiated BM-DC from AhR^–/–^ mice was not completely abrogated, the motility was significantly lower when compared to WT BM-DC (Fig. 4*F*). Brand et al. have shown that the deletion of E-cadherin on LC changed their typical morphology, without any impact on LC maturation and migration (40). E-Cadherin was found to be a target gene of AhR as well (41, 42). Hence, it might be conceivable that the signaling pathways of AhR regulate APC morphology, maturation, migration, and function differently. Recently it was shown that treatment of human DC with the AhR ligands FICZ and Indoxyl 3-sulfate upregulated CCR7 mRNA and protein levels identifying CCR7 as a target gene for AhR (43). Moreover, FICZ exposure of human DC increased their migration potential (43). However, these effects were ligand specific as it was described for the Treg and T_H_17 differentiation (14), which needs to be considered when AhR is targeted for therapeutic modalities.

It is commonly known that AhR activation in mature CCR7^+^ APC can induce IDO transcription, which modulates murine and human LC maturation (15, 44–46). Further, IDO depletes tryptophan, which is crucial for T cell proliferation (47). AhR^–/–^ mice failed to induce IDO and PD-L1 expression upon UV-B irradiation (Fig. 3 *I* and *J*), pointing to an ablation of the tolerogenic APC phenotype and resulting in reduced immunosuppression and protection from EAE in these mice. Hence, it might be interesting to target AhR directly in cutaneous APC to circumvent the disadvantages of UV-B therapy but still profit from the immune-regulating effects during immunoinflammatory diseases.

Of note, conditional deletion of certain genes in CD11c^+^ cells might lead also to an abrogation of these genes in splenic T cells, as it was seen for the ablation of HIF1α in CD11c^+^ cells (48). However, it is highly likely that AhR activation on APC was the major driving effect for UV-B-mediated immunosuppression during EAE since AhR deletion in CD207^+^ cells showed comparable effects to the results observed in AhR^ΔCD11c^ mice (Figs. 2 and 3) and AhR deletion on T cells had a less prominent effect on EAE severity (*SI Appendix*, Fig. S7).

Though, the effect of AhR activation on T cells, such as T_H_1 or T_H_17 cells, has frequently been discussed (14, 15, 49–51). In line with Quintana and Kimura et al., UV-B irradiation failed to induce Foxp3^+^Helios^+^ Treg in mice with a specific AhR deletion in T cells (*SI Appendix*, Fig. S7*B*) most presumably due to an inefficient Treg generation and differentiation (14, 50). UV-B-irradiation had a minor and not significant effect on EAE perpetuation in mice specifically lacking AhR in T cells (AhR^ΔLck^) (*SI Appendix*, Fig. S7*A*) underlying the crucial role of AhR signaling in cutaneous APC for Treg expansion. However, it might be also conceivable that UV-B irradiation had a direct effect on T cell subsets in the skin upon AhR regulation, which needs to be further analyzed.

Taken together, this study clearly demonstrates that the skin plays an important role in sensing environmental stimuli with impact on the development and progression of autoimmune disorders affecting other organs than the skin. Since human cutaneous APC from MS patients were decreased after UV-B irradiation potentially suggesting an enhanced migratory capacity as well as an upregulated AhR expression (Fig. 5). A specific AhR activation in tissue-resident APC for instance by a topical targeted treatment might be potent enough to induce immunosuppression in individuals with MS. However, more studies are needed to fully delineate the systemic effects of AhR ligands as well as the AhR binding partner during neuroinflammatory diseases.

## Materials and Methods

### Mice.

C57BL/6 (purchased from Janvier-Labs, Cedex, France), AhR^–/–^ (52), AhR^fl/fl^ (53), AhR^ΔCD11c^ (54), AhR^ΔCD207^ (55), AhR^ΔCamkIIα^ (56), AhR^ΔLck^ (57), TCR_MOG_ x IgH_MOG_ (Devic) (29) were used at the age of 4 to 12 wk and housed under specific pathogen-free conditions in microisolator cages. Mice were given chow and water ad libitum and were monitored according to the Score-Sheet within the TVA permit. All animal experiments were performed with the approval of the State Review Board of North Rhine-Westphalia (Germany) according to the German law for animal welfare (Tierschutzgesetz) §8, reference number 84-02.04.2013.A139 and 81-02.04.2019.A089.

### Induction of EAE.

EAE was induced in 8- to 12-wk-old mice as indicated before (58). Disease severity was scored daily using the experimental autoimmune neuritis score ranging from scale 0 to 10 as described before (58). Animals were scored in a blinded fashion by two independent investigators. Disease onset was defined as clinical score >1.

### UV-B Irradiation.

Before irradiation and MOG-immunization, the back skin of mice was shaved and UV-B irradiation was performed as described (1). Details regarding the irradiation protocol are outlined in *SI Appendix, SI Materials and Methods*.

### Histology and Immunofluorescence Staining.

Mouse tissues (brain, spinal cord, skin, lymph nodes) were cryopreserved and cut as described before (58). For hematoxylin and eosin (H&E) as well as luxol fast blue (LFB; Merck) staining tissues were embedded in paraffin after transcardial perfusion with PBS and cut into 3 µm sections. To analyze inflammatory foci and demyelinated areas sections were stained with H&E and LFB using standard methods (58). Details can be found in *SI Appendix, SI Materials and Methods*.

Human biopsy material from patients with MS were obtained from the UV-B phototherapy study from Breuer et al. Clinical characteristics of MS patients can be found in *SI Appendix*, Table S2 (1). All experiments were carried out according to the Declaration of Helsinki and were approved by the ethical committee of the University of Münster Medical School (2012-323-f-S; *n* = 6 subjects). Written informed consent was obtained from all patients prior to inclusion in the study.

### Cell Preparation and Flow Cytometry.

Single-cell suspensions of lymph nodes and CNS (brain and spinal cord) were prepared as described previously (58). Details regarding the used antibodies are outlined in *SI Appendix, SI Materials and Methods*.

### Quantification of AhR Ligand Activity.

The mouse Kit promoter (-2159/-37, from ATG) containing two canonical XRE sequences (GCGTG in position -343 and -140) and the mouse Cyp1a1 promoter containing five XRE sequences (GCGTG and reverse CACGC) were subcloned as described in *SI Appendix, SI Materials and Methods*.

### In Vitro Migration Assays.

Bone marrow cells were isolated from femurs and tibias of adult C57BL/6 and AhR^–/–^ mice as described (59). Details regarding the assay protocol are outlined in *SI Appendix, SI Materials and Methods*.

### RNA Isolation and Quantitative Real-Time PCR (qPCR).

RNA was extracted from snap-frozen tissues or purified cells as well as copy DNA was synthesized as described before (58). All reported mRNA levels were normalized to *actb* and relative mRNA expression was calculated according to the 2^−ΔΔct^ method (60). Primer sequences are depicted in *SI Appendix*, Table S3.

### Statistics.

All values are expressed as means ± SEM. Statistically significant differences were assessed by the Mann–Whitney rank sum test (Figs. 1 *A* and *B*, 2*A*, and 4*F* and *SI Appendix*, Figs. S4*E*, S6 *A* and *B*, S7*A*, and S8*A*; two independent groups. One treated and one control, normal distribution not required), Student’s *t* test (Fig. 4*E* and *SI Appendix*, Fig. S7*C*; comparing two datasets following a normal distribution), ANOVA on RANKS (*SI Appendix*, Fig. S3*B*; comparing more than two groups and normal distribution not required) or One-Way ANOVA test (Figs. 1 *C*–*E*, 2 *B* and *C*, 3 *B*, *C*, *E*, *H*, and *J*, and 4 *B* and *D* and *SI Appendix*, Figs. S1*A*, S3*A*, S4*B*, S5*B*, S5*D*, and S8 *B* and *C*; comparing more than two groups). The alpha level was set at <0.05 in all cases and SigmaPlot 12.3 or GraphPad Prism 6 were used to analyze, plot and illustrate data.

## Supplementary Material

Appendix 01 (PDF)

## Data Availability

All study data are included in the article and/or *SI Appendix*.
