# Supplementary material for: AhR signaling in skin-resident CD207+ cells is involved in UV-B-induced amelioration of neuroinflammation
Source: Proc Natl Acad Sci U S A. 2025 Sep 2;122(36):e2424009122. doi: 10.1073/pnas.2424009122 (PMC12435267; doi:10.1073/pnas.2424009122)
Supplement: Supplementary file 1 — Appendix 01 (PDF) [file pnas.2424009122.sapp.pdf]

SUPPLEMENTARY INFORMATION

Supplementary Material and Methods

Supplementary Material and Methods 2

1 UV-B irradiation 2

2 Histology and immunofluorescence staining 2

3 Flow cytometry 3

4 Quantification of AhR ligand activity 4

***5 In vitro* migration assays** 4

Supplementary Tables and Figures 5

Suppl. Table 1: Clinical characteristics of MS patients enrolled in the UV-B trial 5

Suppl. Table 2: Sequences of primers used to amplify mouse and human genes 6

Suppl. Fig. 1. UV-B fails to ameliorate EAE in the absence of AhR 7

Suppl. Fig. 2. Flow cytometry and gating strategy of different cell subsets 8

Suppl. Fig. 3. UV-B-induced activation of AhR 9

Suppl. Fig. 4. Langerin+ cell migration is impaired in AhR-deficient mice 10

Suppl. Fig. 5: IL-10 expression in FoxP3$^-$ cell populations is not a mechanism for an interrelationship of AhR and IL-10 11

Suppl. Fig. 6. UV-B irradiation mediated immunomodulatory effects in Devic mice 12

Suppl. Fig. 7. AhR deletion in T cells had a minor effect on UV-B-mediated amelioration of EAE 13

Suppl. Fig. 8. The deletion of AhR in neurons had no impact on the UV-B-induced protection from EAE 14

Supplement references 16

Supplementary Material and Methods

1 UV-B irradiation

Before irradiation and MOG-immunisation, the back skin of mice was shaved. Mice were exposed to UV-B light (wave length 280-350 nm) for 4 consecutive days (150mJ/cm^2/day, which equaled to 2 minutes of irradiation per day) and left untreated for 3 days. Thereafter, mice were left untreated for 3 days before a second irradiation cycle for 4 days started. This procedure was carried out until the end of the experiment. The days of UV-B irradiation are marked by arrows. Devic mice were irradiated using the same UV-B regimen. Irradiation was started at day 21 after birth and carried out until the mice reached a clinical score of 7, which required euthanasia according to the animal welfare guidelines.. For irradiation we used UV light bulbs with emission from 280 nm to 350 nm radiation, which in principle covers both UVB and UV-A radiation. However, the emission maximum of UV-A is 365 nm. Therefore, only small quantities of UV-A are emitted.

2 Histology and immunofluorescence staining

Mouse tissues (brain, spinal cord, skin, lymph nodes) were cryopreserved and cut as described before[1]. Thereafter, sections were incubated overnight at 4° C with the appropriate dilutions of primary antibodies against CD4 (clone RM4-5; Biolegend), IL-17A (clone TC11-8H4; Biolegend, San Diego, CA), Foxp3 (clone FJK-16s, Thermo Fisher Scientific, Darmstadt, Germany), MHCII (NIMR-4, abcam, Cambridge, UK), CD207 (clone 929F3, kindly provided by Dr. S. Saeland, DermImmun, France), GM-CSF (clone MP1-22E9, Biolegend), IFN-γ (clone XMG1.2, Biolegend) and AhR (clone FF3399, directly labelled, Thermo Fisher Scientific). Subsequently, slides were incubated with AlexaFluor (AF)-594- or AF-488 coupled secondary antibodies. In some experiment's nuclei were counterstained with 4'6-diamidino-2-phenylindole (DAPI; Sigma-Aldrich).

For hematoxylin and eosin (H&E) as well as luxol fast blue (LFB; Merck) staining tissues were embedded in paraffin after transcardial perfusion with PBS and cut into 3 µm sections. To

analyze inflammatory foci and demyelinated areas sections were stained with H&E and LFB using standard methods[1]. Subsequently, slides were analyzed on an Olympus BX63 microscope using the cellSens software (Olympus, Münster, Germany) as well as the ImageJ or the cellSens software (Evident, Münster, Germany).

3 Flow cytometry

The expression of cell surface and intracellular markers was analyzed by multicolor flow cytometry on a Gallios or CytoFLEX (Beckman Coulter, Krefeld, Germany) flow cytometer using the Kaluza 2.1 software (Beckman Coulter). For flow cytometry cells were stained in PBS containing 1 % FCS. Intracellular staining of IDO, IL-22, T-bet, CD207, IL-12, IL-17A, AhR, Foxp3, Helios and IFN-γ was performed after cell permeabilization using the Fix/Perm Buffer Set (Biolegend) according to the manufacturer's instructions.

Suppl. Table 1: List of antibodies used for flow cytometry

Name	Clone	Company
CD4	RM4-5	Biolegend
CD11b	M1/70	
CD11c	N418	
CD326	G8.8	
CD80	16-10A1	
CD24	M1/69	
MHC class II	M5/114	
PD-L1	10F.9G2	
CD172α	P84	
CD44	IM7	Milteny
IDO	mIDO-48	Biolegend
IL-22	Poly5164	
T-bet	4B10	Milteny
CD207	caa8-28H10	
IL-12	REA136	
IL-17a	TC11-18H10.0	
AhR	4MEJJ	Thermo Fisher Scientific
Foxp3	FJK-16s	

Helios	22F6	
IFN-γ	XMG1.2	

4 Quantification of AhR ligand activity

The mouse Kit promoter (-2159/-37, from ATG) containing two canonical XRE sequences (GCGTG in position -343 and -140) and the mouse Cyp1a1 promoter containing five XRE sequences (GCGTG and reverse CACGC) were subcloned in a luciferase reporter plasmid (pGL3 basic, Promega) and obtained from C. Esser (Düsseldorf)[2]. The empty vector pGL3 was used as a negative control according to Kiss et al.[2]. $5x10^4$ Hepa1.6 cells were plated and co-transfected with the pGL3 vector containing the mouse *Cyp1a1* promotor as described previously[2]. After 24 hours medium was replaced and the cells were stimulated with murine serum samples from UV-B irradiated and non-irradiated WT mice. Luciferase activities were determined using the Dual-7 Luciferase Reporter Assay system (Promega) in a Multi-Bioluminate LB 9505C (Berthold Technologies) after 24 hours of stimulation. Firefly luciferase activity (from the reporter plasmids) was normalized to Renilla luciferase activity (from pRL-SV40) according to Kiss et al.[2].

5 In vitro migration assays

Bone marrow cells were isolated from femurs and tibias of adult C57BL/6 and $AhR^{-/-}$ mice as described[3]. Subsequently, single cell suspensions were prepared and cultured in the presence of 5% GM-CSF and IL-4. At day 6 of culture, cells were stimulated either with 0.1 µg/ml Lipopolysaccharide (LPS) (Sigma-Aldrich, Taufkirchen, Germany) or 1.25 mJ/cm^2 UV-B and incubated further for 36 h. At day 9 of culture, cells were collected and used for migration analyses. Real-time and label-free monitoring of haptotactic cell migration was carried out with the xCELLigence system and RTCA software version 2.0.0.1301 (Roche, Mannheim, Germany). To this end, the filter membranes of CIM plates were coated from below with 10 µg/ml bovine collagen I in 5 mM acetic acid over night at 4 °C. Both bottom and top compartments were filled with RPMI medium containing 10 mM 4-(2-hydroxyethyl)-1-

piperazineethanesulfonic acid (HEPES) (Sigma-Aldrich) and 2.5 x 10^4 cells were added to the top compartment of each well. Cell migration was monitored for 24 h at 37 °C in a humidified incubator at 5 % CO2, and migration in the time window of 3.5 - 8.5 h was quantified as cell index and after normalization to the values at start as a delta cell index. Cell index represents the impedance value between the electrodes that are located at the bottom site of the filter. Impedance rises as migrating cells reach the electrodes, attach and spread there.

Supplementary Tables and Figures

Suppl. Table 2: Clinical characteristics of MS patients enrolled in the UV-B trial

No. of patients	*Age (yr)*	*Sex*	*MS (first manifestation, age in years)*	*Treatment*	*Skin type*	*MSFC* before UVB-therapy*	*MSFC* after UVB-therapy*
2	41.5	F	37.5	Interferon-β 1a	I,III	0.51	0.74
1	48	M	34	Interferon-β 1a	II	-0.22	0.13
2	44	M	24.5	Interferon-β 1b	I,II	1.66	0.73
1	52	F	31	Natalizumab	II	-0.3	-0.1
2	39	F	30	Glatiramer acetate	II	-1.22	-1.37
2	57	F	41.5	/	III, II	0.3	0.21
1	52	M	45	/	II	0.31	0.15

*MSFC = Multiple sclerosis functional composite (multidimensional, three component scale to assess impairment in MS patients).

Patients from the grey columns were used for immunofluorescence staining.

Suppl. Table 3: Sequences of primers used to amplify mouse and human genes

Gene	sense (5'→ 3')	anti-sense (5'→ 3')
**MOUSE GENES:**		
Actb	TTGCTGACAGGATGCAGAAC	TGATCCACATCTGCTGGAAG
Ahr	AGACCGGCTGAACACAGAGT	CTTGCAAATCCTGCCAGTCT
Ahrr	GGGTAAAGAGCTTCTTCCAAGC	ACGGGGAACCCTCTGTATG
Cyb1a1	CCCCCCAAAACAAAAACAGAT	AGAGTGTGCTAATATTTCACAGGTCAA
Cyb1b1	GGTTAACCATGACCGGGAACT	TGCCCAAACCAAAGAGAGTGA
Ido1	GAGTCTTGATGTCCTTCTGG	CTACTATTGCGAGGTGGAAC

Suppl. Fig. 1. UV-B fails to ameliorate EAE in the absence of AhR

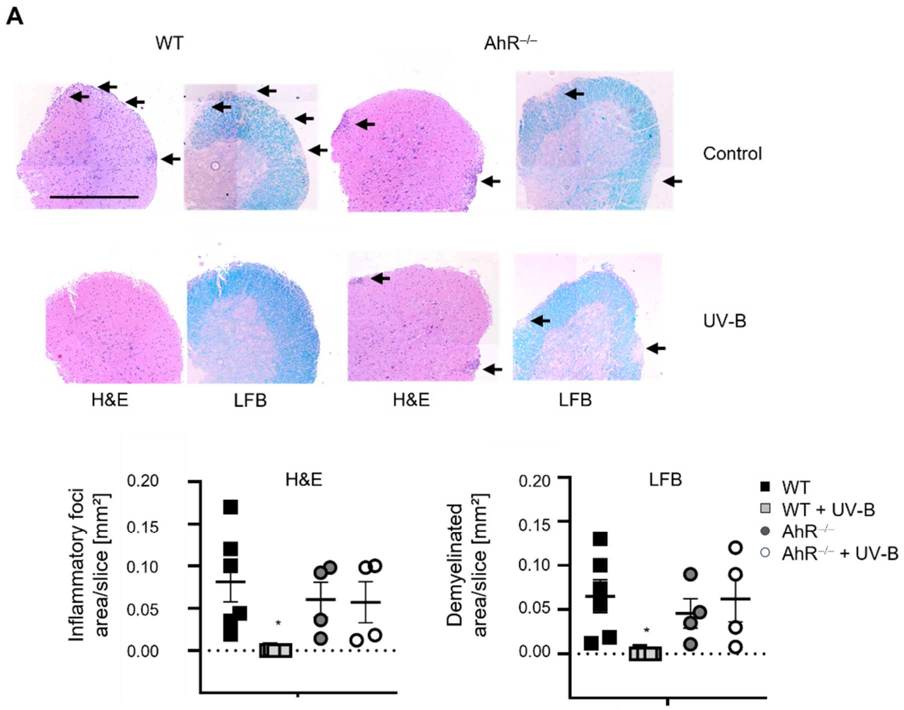

(**A**) Representative hematoxylin and eosin (H&E) as well as Luxol fast blue (LFB) staining in lumbal spinal cord at disease maximum. Original magnification 100 x, scale bar = 200 µm. Infiltration of mononuclear cells and demyelination are indicated by black arrows. Statistical analyzes of inflammatory foci and demyelinated areas from *n* ≥ 3 mice from three different experiments are shown (*P < 0.05 versus non-irradiated controls tested with One-way ANOVA. P-value was adjusted with Tukeys test for multiple comparisons).

Suppl. Fig. 2. Flow cytometry and gating strategy of different cell subsets

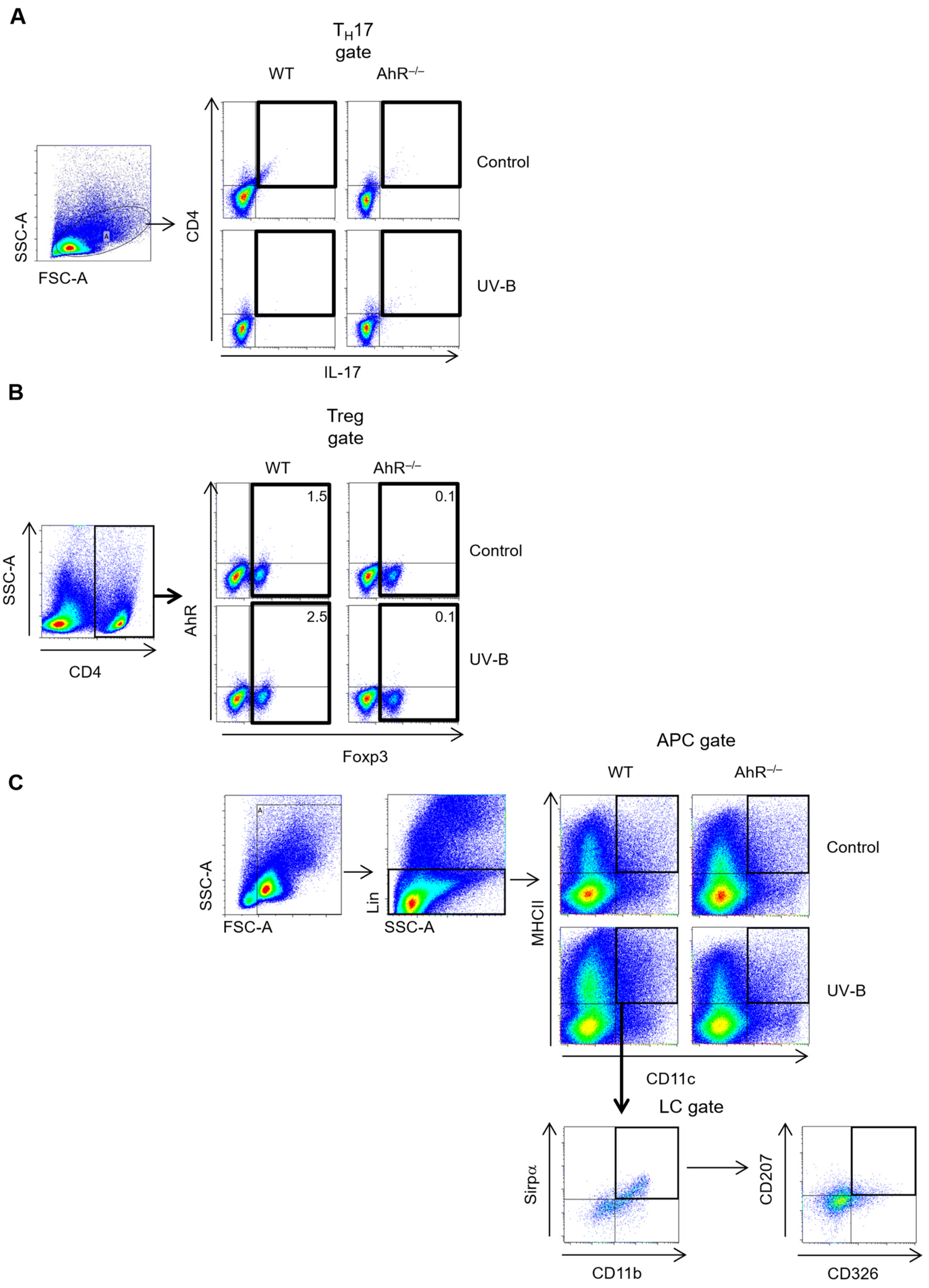

(**A**) Gating strategy of T_H17 cells in the skin draining lymph nodes at disease maximum. Representative density plots are shown. Cells are gated for lymphocytes and $CD4^+IL\text{-}17^+Ror\text{-}\gamma t^+$. **(B)** Gating strategy of Treg in skin draining lymph nodes at disease maximum.

Representative density plots are shown. Cells are gated for lymphocytes and CD4^{+}Foxp3^{+}. **(C)** Gating strategy of APC and LC in skin draining lymph nodes at disease maximum. Representative density plots are shown. Lineage (Lin) antibodies include CD3 (T cells) and CD19 (B cells). Cells are gated for Lin^{-}MHCII^{+}CD11c^{+} for APC and Lin^{-}MHCII^{+}CD11c^{+}CD11b^{+}Sirpα^{+}CD207^{+}CD326^{+} for LC according to Clausen et al.[37]

Suppl. Fig. 3. UV-B-induced activation of AhR

A

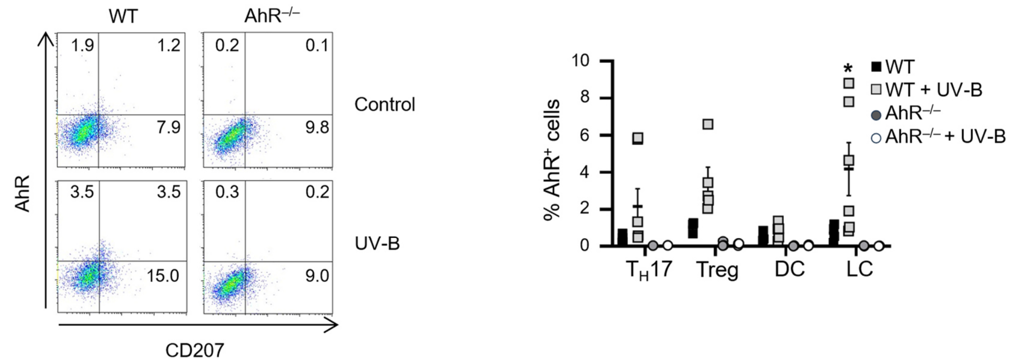

B

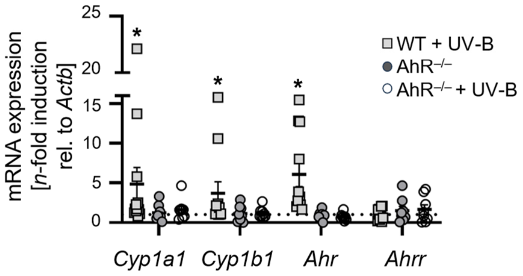

(**A**) Flow cytometry of AhR expressing cells in the skin-draining lymph nodes at disease maximum. Representative density plots **(left)** and statistics from $n \geq 3$ mice per group **(right)** are shown (*P < 0.05 versus non-irradiated controls tested with One-way ANOVA. P-value was adjusted with Dunnett´s test for multiple comparisons). Gating strategy is depicted and visualized in Suppl. Fig. 2. **(B)** mRNA expression in murine skin from $n = 8$ MOG-immunized mice at disease maximum with or without UV-B treatment (*P < 0.05 versus non-irradiated controls (dotted line) tested with ANOVA on RANKS).

Suppl. Fig. 4. Langerin+ cell migration is impaired in AhR-deficient mice

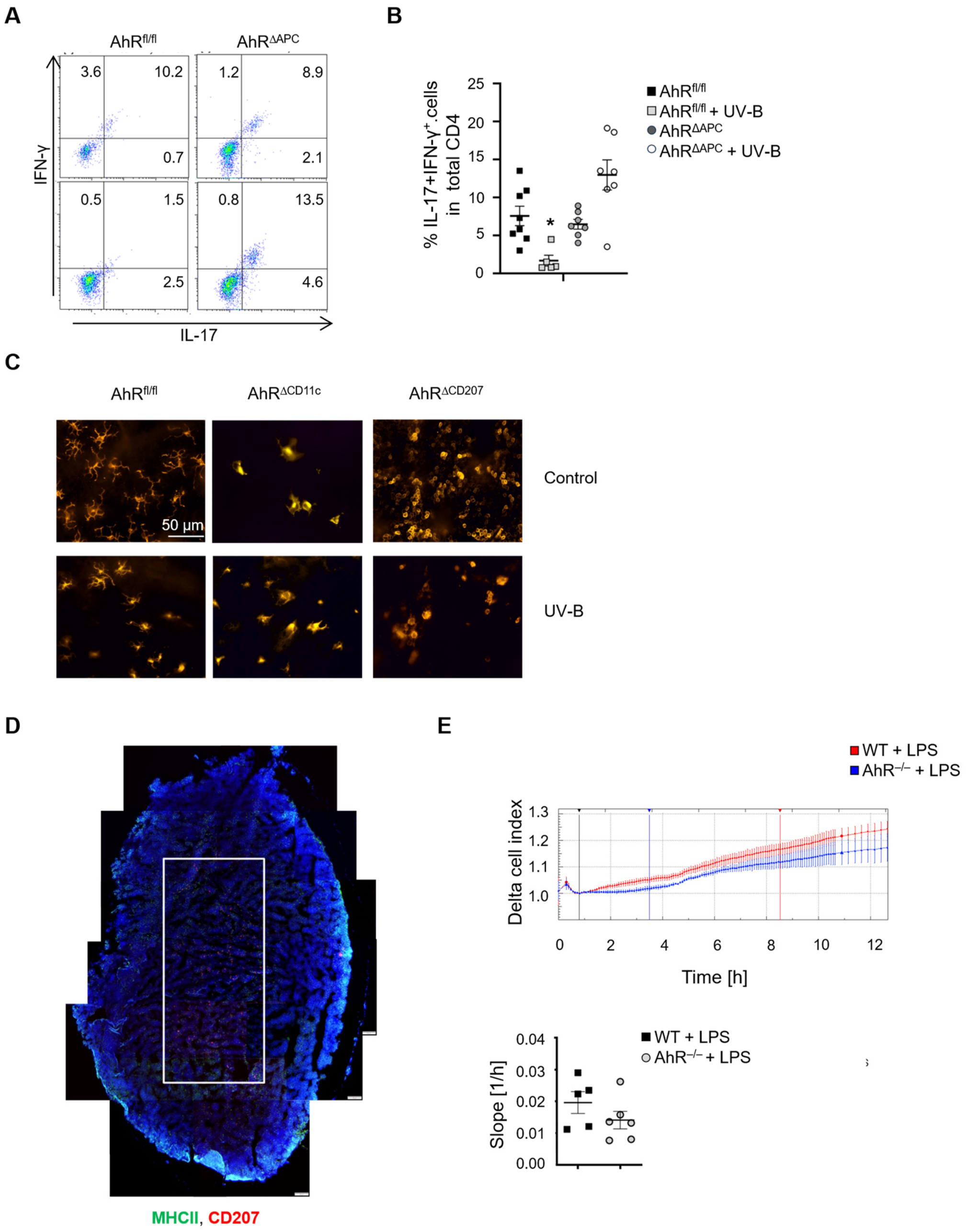

(A□B) Flow cytometry analyzes of CD4$^+$ T cells expressing IL-17 and IFN-γ in the CNS at disease maximum. Representative density plots **(A)** and statistics **(B)** from $n \geq 5$ mice per group are shown (*P < 0.05 versus non-irradiated controls tested with One-way ANOVA. P-value was adjusted with Tukey's test for multiple comparisons); IFN-γ$^+$ and IL-17$^+$ cells were gated for CD4$^+$CD44$^+$ T cells and intracellular was performed after cell permeabilization. **(C)** Immunofluorescence staining of murine epidermal sheets from UV-B irradiated and non-irradiated AhR$^{fl/fl}$, AhR$^{\Delta CD11c}$ and AhR$^{\Delta CD207}$ mice at disease maximum. One representative

image is shown. Original magnification 200 x, scale bar = 50 µm. **(D)** Immunofluorescence staining of mouse skin draining lymph nodes at disease maximum. One representative image is shown. CD207⁺ cells are mainly in the paracortical structure. Cells are counterstained with DAPI. **(E)** Migration assay of LPS-stimulated BM-DC from WT and AhR$^{-/-}$ mice. Cells were generated in the presence of GM-CSF and IL-4 and stimulated with 0.1 µg/ml LPS. Cells were seeded on a CIM plate coated with 10 µg/ml bovine Collagen-I. Cell migration was measured by impedance and read out as Delta cell index. Migration rates are measured as slopes of delta cell index values over a time period (marked by blue and red vertical lines. Representative migration of BM-DC of WT and AhR$^{-/-}$ mice (left) as well as statistics (right) during 5 h of measurement from *n* = 2 mice and 5-6 replicates are represented (*P < 0.05 versus WT controls tested with Mann-Whitney Rank Sum Test).

Suppl. Fig. 5: IL-10 expression in FoxP3^{-} cell populations is not a mechanism for an interrelationship of AhR and IL-10

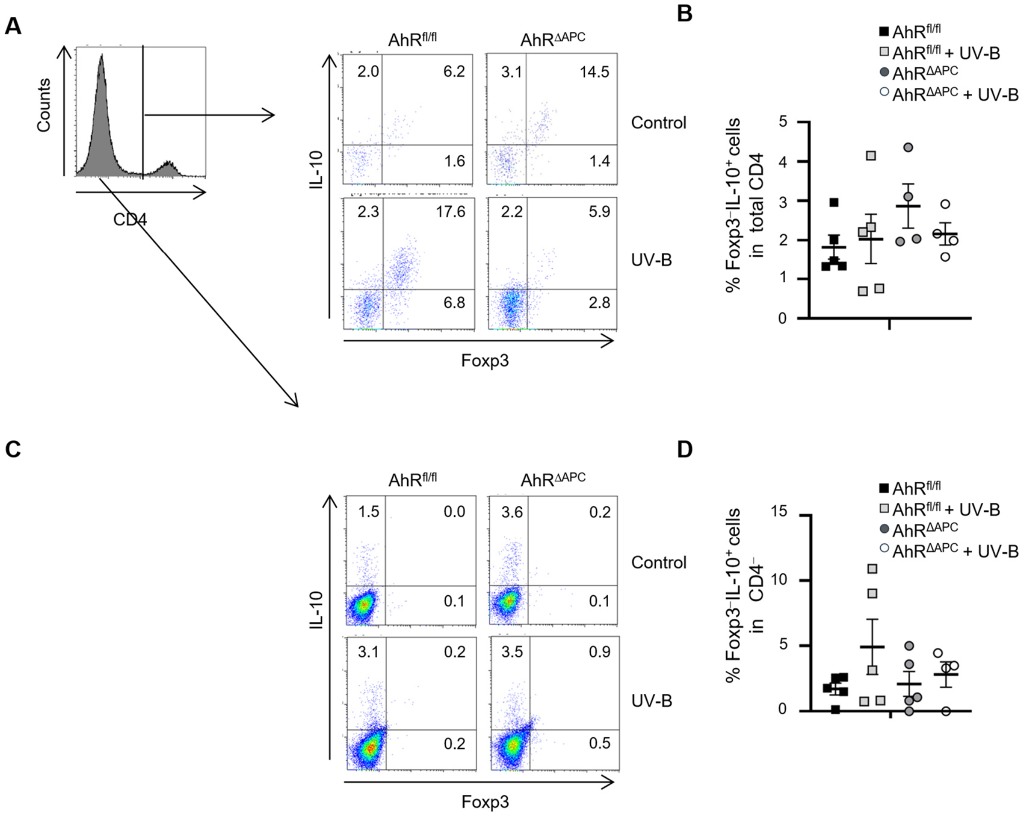

(A - D) Flow cytometry analyzes of IL-10 expression in Foxp3⁻ cell populations in the CNS at disease maximum. Cells are gated for CD4⁺ or CD4⁻ **(left)**. Representative density plots **(A□C)** and statistics **(B□D)** from *n* ≥ 4 mice per group are shown (*P < 0.05 versus non-irradiated

controls tested with One-way ANOVA. P-value was adjusted with Tukey's test for multiple comparisons); Intracellular was performed after cell permeabilization.

Suppl. Fig. 6. UV-B irradiation mediated immunomodulatory effects in Devic mice

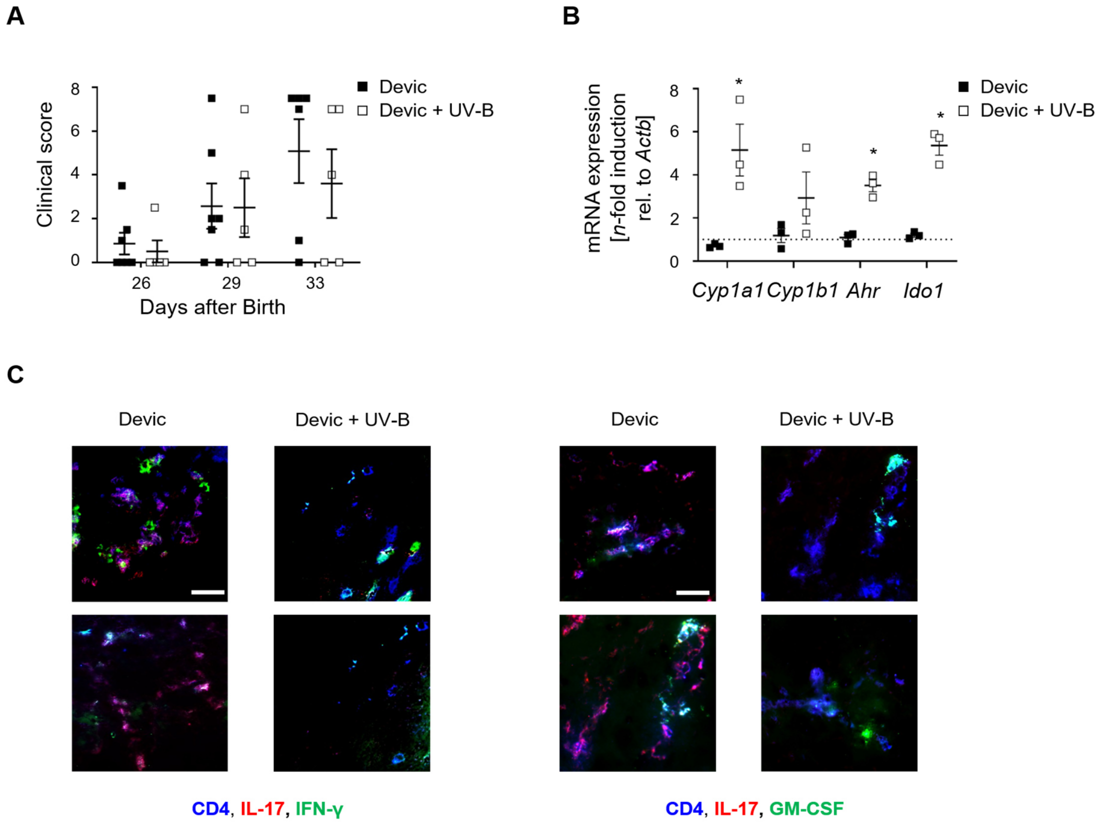

(**A**) 21 days after birth, Devic mice (*n* = 5–7) were irradiated with 150mJ/cm^2/day UV-B light as described in the methods. Mean clinical scores are shown. **(B)** mRNA expression in murine skin from *n* = 3 Devic mice at disease maximum with or without UV-B treatment (*P < 0.05 versus non-irradiated controls (dotted line) tested with Kruskall-Wallis Test).**(C)** Immunofluorescence staining using antibodies against CD4 (blue), IL-17 (red), IFN-γ (green) and GM-CSF (green) in lumbar spinal cord from irradiated and non-irradiated Devic mice at d33 after birth. Two representative image from n = 1 -3 mice is shown. Scale bars represent 20 µm, original magnification = 400x.

Suppl. Fig. 7. AhR deletion in T cells had a minor effect on UV-B-mediated amelioration of EAE

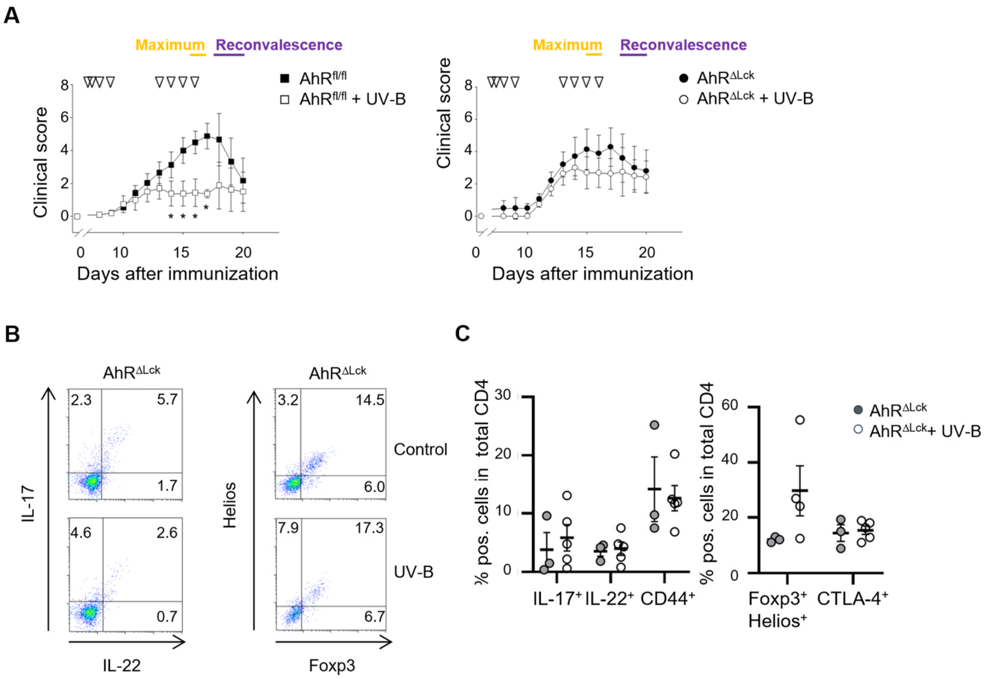

AhR$^{fl/fl}$ (n = 8 at disease maximum/n = 3 at reconvalescence) and AhR$^{\Delta Lck}$ (n = 7 at disease maximum, n = 5 at reconvalescence) mice were immunized with MOG peptide and irradiated with 150mJ/cm^2/day UV-B light as described in the methods. ∇= triangles indicate the days of irradiation. **(A)** Mean clinical scores are shown (*P < 0.05 versus non-irradiated controls tested with Mann-Whitney Rank Sum Test). **(B□C)** Flow cytometry of effector T cells **(left)** and Treg **(right)** in the CNS at disease maximum. Representative density plots **(B)** and the statistical evaluation from n = 3 mice per group **(C)** are shown (*P < 0.05 versus non-irradiated controls tested with Student's t-Test). IL-17$^+$ and IL-22$^+$ cells are gated for CD4$^+$CD44$^+$T cells and staining was performed after cell permeabilization.

Suppl. Fig. 8. The deletion of AhR in neurons had no impact on the UV-B-induced protection from EAE

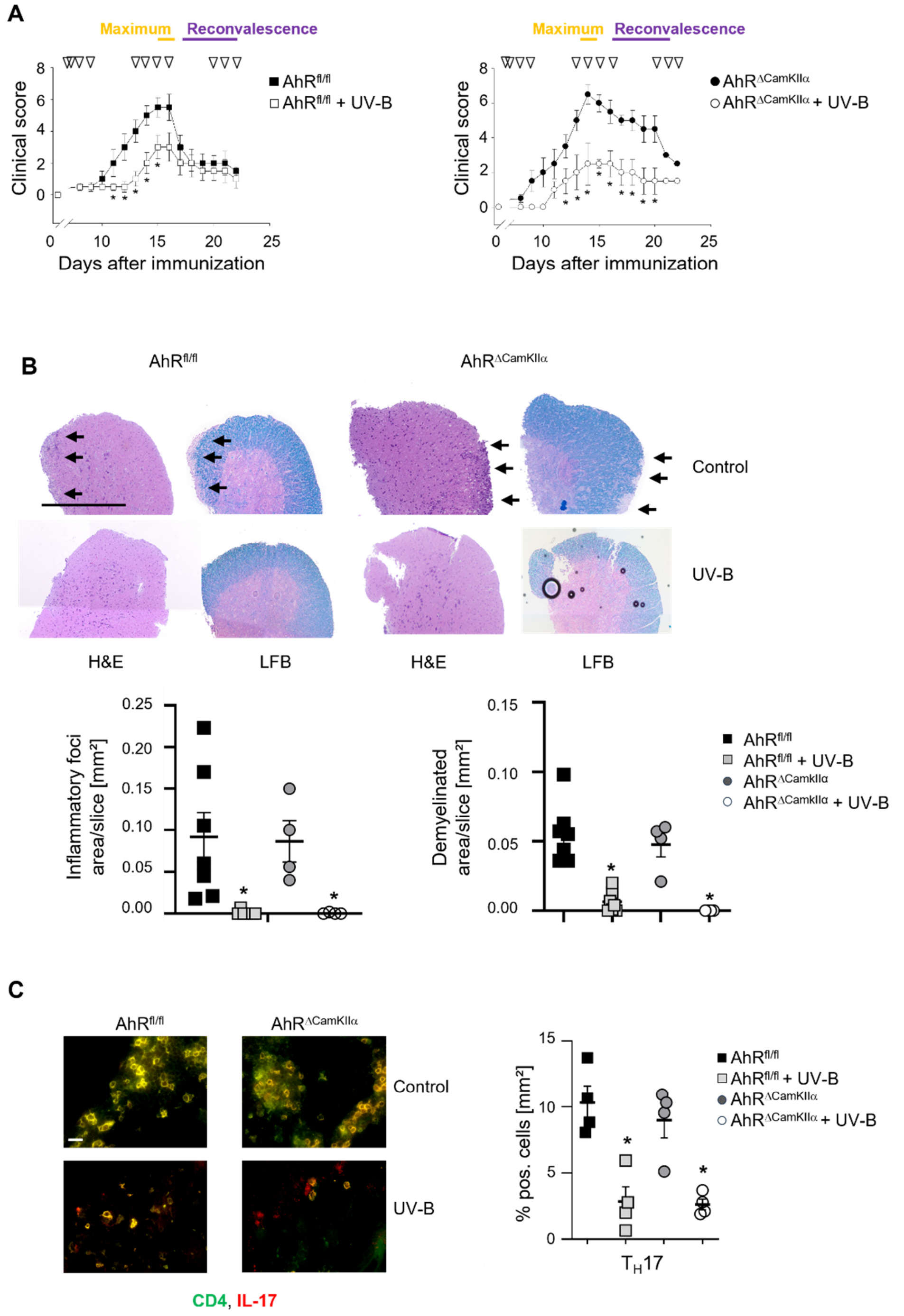

AhR$^{fl/fl}$ (n = 9 at disease maximum)/n = 3 at reconvalescence) as well as AhR$^{\Delta CamKII\alpha}$ (n = 12 at disease maximum, n = 3 at reconvalescence) mice were immunized with MOG peptide and irradiated as described before. **(A)** Mean clinical scores are shown (*P < 0.05 versus non-irradiated controls tested with Mann-Whitney Rank Sum Test). **(B)** Representative H&E as well

as LFB staining in lumbal spinal cord at disease maximum as well as statistical analyzes of inflammatory foci and demyelinated areas from $n \geq 3$ mice out of three different experiments are shown. Original magnification 100 x, scale bar = 200 µm. Infiltration of mononuclear cells and demyelinated areas are indicated by arrows (*P < 0.05 versus non-irradiated controls tested with ANOVA. P-value was adjusted with Tukeys test for multiple comparisons). **(C)** Immunofluorescence staining of murine brain tissue from UV-B irradiated and non-irradiated $AhR^{fl/fl}$ and $AhR^{\Delta CamkII\alpha}$ mice at disease maximum. One representative image **(left**) as well as the statistical evaluation from $n \geq 4$ mice out of three different experiments **(right)** are shown. To evaluate T_H17 or Treg numbers, cells were counted in 9 visual fields of each cryosection. Original magnification 400 x, scale bar = 20 µm (*P < 0.05 versus non-irradiated controls tested with One-way ANOVA. P-value was adjusted with Tukeys test for multiple comparisons).

Suppl. Fig. 9. Schematic overview about the proposed mechanism how UV-B mediated AhR activation regulates CD207+ APC maturation and migration resulting in Treg expansion and amelioration of EAE

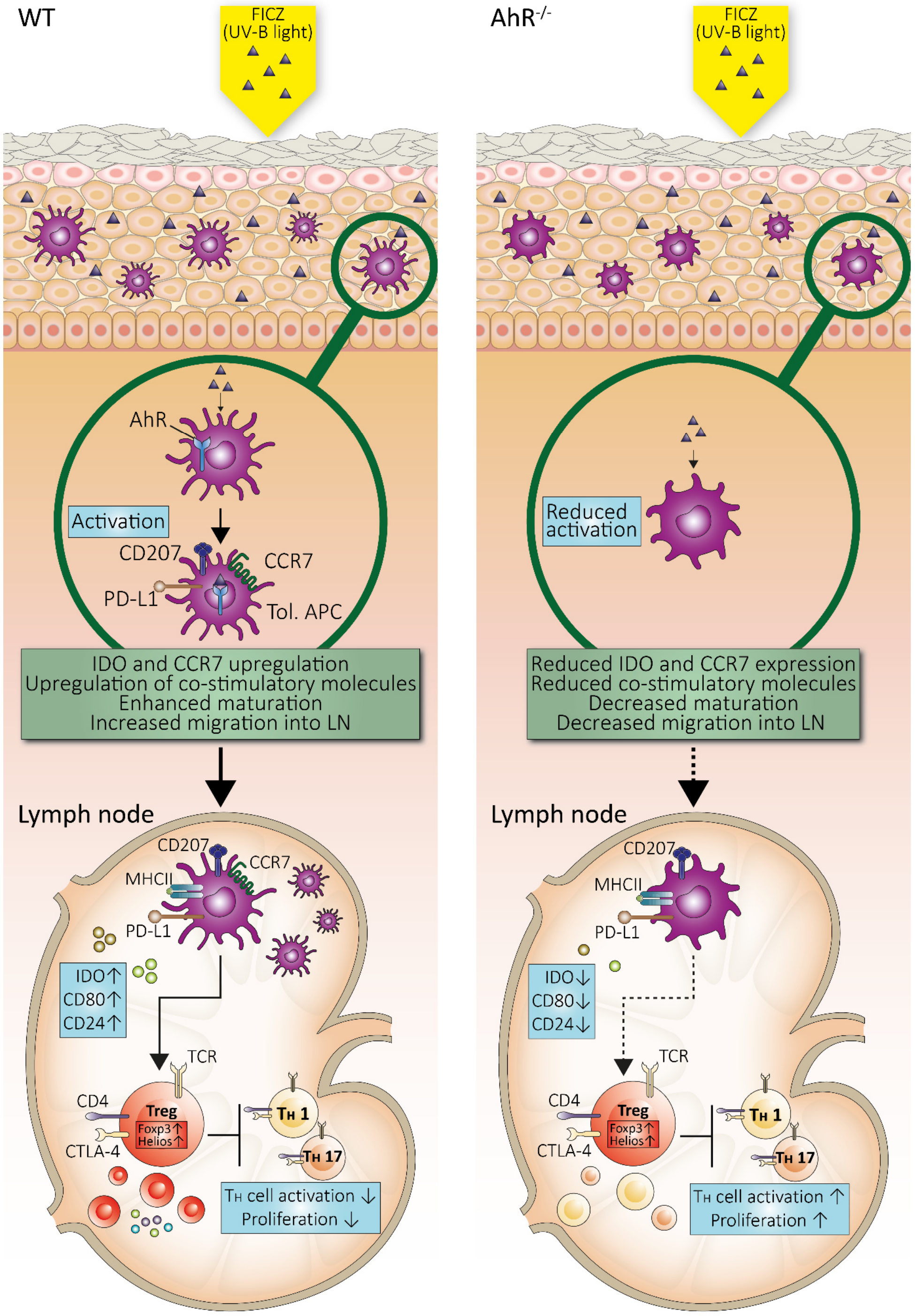

In WT mice AHR activation by UV-B irradiation resulted in the upregulation of IDO and co-stimulatory molecules and therefore, an enhanced maturation of APC. Mature cutaneous CD207^{+}CCR7^{+} APC migrated into the lymph nodes, expanded Treg and thereby, suppressed

pathogenic T cells during MOG-induced EAE. APC lacking AhR were less granular and showed a reduced expression of IDO, CCR7 and co-stimulatory markers upon UV-B irradiation. APC were less mature with a lower capacity to migrate to the draining lymph nodes resulting in the abrogation of UV-B-mediated Treg expansion and suppression of pathogenic effector T cells during MOG-induced EAE.

Supplement references

1. Mykicki, N. *et al*. Melanocortin-1 receptor activation is neuroprotective in mouse models of neuroinflammatory disease. *Sci Transl Med* **8**, 362ra146 (2016).

2. Kiss, E.A. *et al*. Natural aryl hydrocarbon receptor ligands control organogenesis of intestinal lymphoid follicles. *Science* **334**, 1561-1565 (2011).

3. Auriemma, M. *et al*. α-MSH-stimulated tolerogenic dendritic cells induce functional regulatory T cells and ameliorate ongoing skin inflammation. *J Invest Dermatol* **132**, 1814-1824 (2012).
